# Evaluation of the Clinical and Cost Effectiveness of Intermediate Care Clinics for Diabetes (ICCD): A Multicentre Cluster Randomised Controlled Trial

**DOI:** 10.1371/journal.pone.0093964

**Published:** 2014-04-15

**Authors:** Andrew Wilson, Joseph Paul O’Hare, Ainsley Hardy, Neil Raymond, Ala Szczepura, Ric Crossman, Darrin Baines, Kamlesh Khunti, Sudhesh Kumar, Ponnusamy Saravanan, the C C D trial group

**Affiliations:** 1 Department of Health Sciences, University of Leicester, Leicester, United Kingdom; 2 Warwick Medical School, University of Warwick, Coventry, United Kingdom; 3 WISDEM centre, University Hospital Coventry and Warwickshire, Coventry, United Kingdom; 4 Academic Centre for Diabetes, Endocrinology and Metabolism, George Eliot Hospital, Nuneaton, United Kingdom; Postgraduate Medical Institute & Hull York Medical School, University of Hull, United Kingdom

## Abstract

**Background:**

Configuring high quality care for the rapidly increasing number of people with type 2 diabetes (T2D) is a major challenge worldwide for both providers and commissioners. In the UK, about two thirds of people with T2D are managed entirely in primary care, with wide variation in management strategies and achievement of targets. Pay for performance, introduced in 2004, initially resulted in improvements but disparities exist in ethnic minorities and the improvements are levelling off. Community based, intermediate care clinics for diabetes (ICCDs) were considered one solution and are functioning across the UK. However, there is no randomised trial evidence for the effectiveness of such clinics.

**Trial Design, Methods and Findings:**

This is a cluster-randomised trial, involving 3 primary care trusts, with 49 general practices randomised to usual care (n = 25) or intervention (ICCDs; n = 24). All eligible adult patients with T2D were invited; 1997 were recruited and 1280 followed-up after 18-months intervention. Primary outcome: achievement of all three of the NICE targets [(HbA1c≤7.0%/53 mmol/mol; Blood Pressure <140/80 mmHg; cholesterol <154 mg/dl (4 mmol/l)]. Primary outcome was achieved in 14.3% in the intervention arm vs. 9.3% in the control arm (p = 0.059 after adjustment for covariates). The odds ratio (95% CI) for achieving primary outcome in the intervention group was 1.56 (0.98, 2.49). Primary care and community clinic costs were significantly higher in the intervention group, but there were no significant differences in hospital costs or overall healthcare costs. An incremental cost-effectiveness ratio (ICER) of +£7,778 per QALY gained, indicated ICCD was marginally more expensive at producing health gain.

**Conclusions:**

Intermediate care clinics can contribute to improving target achievement in patients with diabetes. Further work is needed to investigate the optimal scale and organisational structure of ICCD services and whether, over time, their role may change as skill levels in primary care increase.

**Trial Registration:**

ClinicalTrials.gov NCT00945204; National Research Register (NRR) M0014178167.

## Introduction

The burden of diabetes is increasing rapidly across the world, including low and middle-income countries [1]. More than 370 million people live with diabetes world-wide and 90% of them have type 2 diabetes (T2D). Nearly 5 million people died due to diabetes in 2012 alone and 50% of these deaths happened in people under the age of 60 years [1]. In the United Kingdom (UK), cardiovascular mortality in middle-aged people (40–65 years of age) is 3 times higher if they have diabetes, despite apparent improvement in their risk factors [2]. Among the non-communicable diseases (NCDs), diabetes is the only NCD where the projected morbidity and mortality is expected to increase [3]. Configuring high quality, evidence based and patient centred services that can prevent higher cardiovascular mortality in all patients with diabetes is a major challenge and a perfect model has been elusive for commissioners and providers of care [4].

The cost burden of T2D is also very high and more than 470 billion US dollars (USD) were spent in 2012 across the world, with the majority of spending in high-income countries [1]. Over the past two decades much emphasis has been placed on improving the ways of delivering diabetes care, at reduced cost, whilst maintaining or improving quality [5]. “Case finding” approaches [6], [7], “care closer to home” and “pay for performance” [4] are such examples. Prior to the introduction of pay for performance as a part of the Quality and Outcomes Framework (QoF) in 2004, wide variation existed in the care of patients with T2D in primary care including referral to specialist services [8]. Although this incentive improved overall process and intermediate outcome measures, significant disparity still exists in ethnic minorities [9]. The improvements are also levelling off, which may partly be due to less challenging targets to secure the QoF points for pay for performance [10].

Nationwide audit data for England 2009–10, showed that more stringent targets for glycosylated hemoglobin (HbA1c≤7.5%/58.5 mmol/L), blood pressure (BP<140/80 mmHg) and total cholesterol (<154 mg/dl/<4.0 mmol/l) were achieved in only 67%, 69% and 41% of people with T2D. Poor glycaemic control was associated with younger age and social deprivation [4]. There were still significant variations between general practices, with practices in areas of high deprivation and serving populations with higher proportions of ethnic minorities less likely to achieve adequate levels of control, [9], [11], [12] as were practices with lower levels of organisation [13].

In an attempt to improve diabetes care and due to the uncertainty over the cost-effectiveness of hospital based specialist services, alternative services to support primary care have been commissioned widely across the UK. These clinics are called “Intermediate Care Clinics for Diabetes” (ICCD). Typically these are community based, multidisciplinary teams, working closely with general practices. Recent evidence has suggested that the most effective interventions include team changes and case management [6]; it is anticipated that such new services may deliver high quality care nearer to the patients, potentially at a lower cost. In some instances, evaluations of such clinics have been conducted [14]–[16], but none has been evaluated in a randomised trial or been comprehensive enough to include both economic alongside clinical evaluation. The objective of the current study is to evaluate the clinical and cost effectiveness of the ICCDs based in three primary care trusts (PCTs) in England in a cluster randomised controlled trial. Our findings show that such clinics may improve cardiovascular risk factors and provide care closer to home without any increase in overall costs.

## Methods

The protocol for this trial and supporting CONSORT checklist are available as supporting information; see Checklist S1 and Protocol S1.

### Ethics Statement

The study protocol was approved by Trent Multi-centre Research Ethics Committee (REC 06/MRE04/41). Institutional review boards (Leicester, Coventry and Nuneaton) provided research governance approval at each site. All protocol amendments were approved by the REC. The trial was registered before participants were recruited in the National Research Register [NIHR NRR id: M0014178167; 29^th^ Oct 2007] and subsequently in the international trial register [ClinicalTrials.gov: Identifier NCT00945204; 23^rd^ July 2009]. A detailed protocol has been published elsewhere [17].

### Study Design and Participants

The study design was a pragmatic two-arm cluster randomised controlled trial, conducted in three English PCTs in the East and West Midlands. All were in urban areas with a higher than average prevalence of diabetes and serving ethnically diverse populations. At each site, ICCD clinics operated for an 18-month period, the first starting in September 2008. Practices recruited to the study were randomised (in-house software) by the UK Clinical Research Network (UKCRN) accredited Warwick Clinical Trials Unit to either control (usual care) or intervention. The intervention practices had access to ICCD clinics. Practices randomised to control were reminded of local diabetes management guidelines and continued to manage their patients, including hospital referrals, in the usual manner. Recruitment of practices and patients started between Jan 2008 and May 2009 and the baseline assessment were completed between Oct 2009 and Jan 2010, in the three centres. The follow up were completed between Aug 2010 and Sep 2011.

All general practices in participating PCTs were invited to take part. Randomisation was undertaken by an independent clinical trials unit after written agreement had been obtained. Randomisation was stratified by practice size and PCT to achieve balanced intervention and control arms. Participants were adults aged 18 years or over, diagnosed with T2D, with no severe cognitive impairment, no severe mental illness and not receiving terminal care. Apart from large practices, where the number invited was capped at 200, all eligible patients were approached by a letter from their general practitioner (GP) enclosing the study information sheet with reply cards. To enhance participation rates, GPs and practice nurses were also asked to introduce eligible patients to the study opportunistically when they attended for a consultation.

### Clinical and Cost Outcome Measures

The primary outcome measure (combined control) was the proportion of participants reaching all three of the targets (HbA1c, BP and total cholesterol) recommended in NICE guidelines (National Institute for health and Clinical Excellence) for patients with T2DM [18]. These targets were: BP<140/80 mmHg, total cholesterol <154 mg/dL (<4 mmol/l) and HbA1c<7.0% (53 mmol/mol). For HbA1c, the guidelines recommend a ‘general’ target of 6.5% (48 mmol/mol), but with flexibility for individual patients aimed to be between 6.5–7.5% (48–58 mmol/mol); after discussion, the project steering group set the target at 7.0% (53 mmol/mol).

Secondary outcomes comprised the proportion of participants reaching targets for individual risk factors (HbA1c, BP and total cholesterol), plus magnitude of changes in these, and health related quality of life (HRQoL) measured using the Euro-QoL 5-dimensions (EQ-5D) questionnaire [19]. The incremental cost effectiveness of the intervention was estimated.

### Patient Assessment Procedures

An independent research nurse or research assistant conducted patient assessments using standard operating procedures after obtaining written informed consent. Detailed history including demographic data was collected at baseline. Assessments took place throughout the day and most were conducted in the patients’ primary care practices. Patients were also offered evening appointments at more convenient central locations. Baseline examinations included BP using an average of three readings from an automated sphygmomanometer, height and weight (BMI) and self-reported smoking. Assays for cholesterol and HbA1c were sent to the laboratory normally used by the practice and reported in the same way as other samples from the practice. Blood tests were not repeated if a result from the previous three months was available from the GP records. All test results were made available to the GP.

Emotional functioning using the ‘Problem Areas in Diabetes’ (PAID) questionnaire [20] continuity of care using the ‘Continuity of Care Questionnaire’ [21]. The study included a qualitative evaluation to be published separately.

Follow up assessments measured the same variables, and also included information on resource use for the economic analysis. In cases where patients did not attend follow up, we used the last reading in the GP record for HbA1c, BP and cholesterol if these had been assessed within three months of the planned follow up. It was not feasible for researchers to be blinded to allocation, but all outcome measures were objective and laboratory staff blind to participants’ randomisation status.

### The Intervention

**Figure 1:**
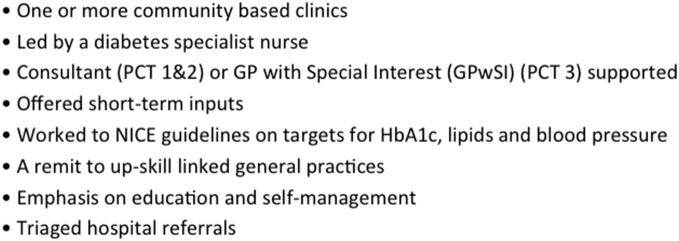
Key features of the intermediate care services.

Common features of ICCDs are listed in Figure 1. Clinics worked closely with hospital-based specialist teams and community services, including podiatry and dietetic services. Guidelines for referral to the ICCDs were common across all sites, and included people with poorly controlled T2DM and poorly controlled cardiovascular risk factors. Patients were managed by the ICCD team until control of risk factors was achieved and then referred back to primary care. In PCTs 1 and 2, only trial participants were eligible to attend the clinics. In PCT 3, the clinics were available to all patients in the intervention practices, whether or not they agreed to participate in the trial, at the request of commissioners.

### Sample Size

To detect a difference of percentage well controlled from 50% in control group to 60% in intervention group (alpha = 0.05 Power = 0.8) not allowing for clustering requires a sample size of 408 subjects in each arm. Using an Intra-cluster correlation coefficient (ICC) of 0.047, and with 72 patients in each cluster, the necessary sample size in each arm was 1,770, a total of 3,540. This number is also adequate to detect a 10% difference in cholesterol control (from 60% to 70%) and blood pressure control (from 60% to 70%). Estimates of ICC for blood pressure and cholesterol were taken from the UK Asian Diabetes Study (UKADS), a study of care provision for people of South Asian ethnicity with diabetes [22]. Assuming the ICC for our primary outcome (combined control of HbA1c, blood pressure and cholesterol) was 0.05 and achievement (from a baseline of 15–20% as suggested by local audit data) was at 20% in the control arm and 30% in the intervention arm, we would need a total of 2,848 patients. In summary, the planned sample size was adequately powered to detect differences in control of the three individual outcomes, and well powered to detect differences in the combined outcome.

Although the study was successful in recruiting 49 practices (11, 13 and 25 in PCT 1, 2 and 3 respectively), recruitment of patients was lower than expected. In total 1997 patients were recruited, with an average of 42 per practice. When recruitment was complete, we recalculated power assuming 75% follow up as reported by UKADS [22]. This showed that the trial had 80% power to detect a 12% difference in the primary outcome measure (combined control). These revisions were reported to and agreed by the funding body.

### Analysis

Intention-to-treat (ITT) comparisons of the two groups were conducted, with the primary dependent variable being the proportion of patients achieving the primary outcome (combined control). The main analysis included only patients with data at both baseline and follow-up; patients with baseline assessment but no evidence of further engagement with the study were excluded. Wherever follow-up data was partially present but with some missing values, those values were replaced where possible using last observation carried forward (LOCF). The LOCF values generated for the outcome variables were then studied to check they did not result in measures at either extreme of the distribution: these checks revealed that the distribution of the LOCF values closely resembled those of the variables themselves. The fact that some patients present at baseline were excluded from the analysis introduces the possibility of bias.

Unadjusted chi-square test was used to compare baseline characteristics for all the descriptive measures. Analyses used a mixed effects logistic regression model, and adjusted for baseline characteristics at both practice and individual level. The mixed effects logistic regression model was constructed including GP practice as a random effect. We included the baseline combined control as a fixed effect plus additional fixed effects; age, gender, ethnicity, smoking status, PCT, deprivation index [23], hypertension, ischaemic heart disease, cerebrovascular disease, heart failure, peripheral vascular disease and renal failure. All variables were retained in the model. Estimates, standard errors and p-value for the intervention effect and other covariates were estimated, along with odds ratios and 95% confidence intervals, adjusting for potential confounding variables and allowing for the effect of the cluster randomisation [24]. The intervention was tested at the 5% (two-tailed) level. All statistical procedures were performed in the R 2.13.0 statistical package, using the lmer function for mixed effect models.

Further analyses examined secondary outcomes; individual variables contributing to combined control, again adjusting for confounders and allowing for the cluster randomisation. We separately analysed the percentage of patients controlling HbA1c, blood pressure (systolic and diastolic), and lipids. These variables were also analysed as numerical measures and ICCs calculated. This required a consideration of whether a normal approximation was valid, using Q-Q plots in each case. Likelihood ratio tests were performed.

### Economic Evaluation

The within trial economic evaluation compared direct costs and 18-month outcomes of patients randomised to intervention versus control. The primary perspective adopted was that of the NHS as commissioner. ICCD costs were estimated in each trial site using a primary costing approach to include staff, accommodation and consumables. In PCT1 and PCT2 clinics were available only to trial participants so all costs were included; in PCT3 the proportion of service costs attributable to trial patients was included in the analysis. Other NHS resource use was recorded via patient questionnaires to include community and hospital resource use. Unit costs per ICCD visit, overall ICCD costs per patient referred to the service, and average total care cost per patient was estimated. Costs were standardised to 2009/10 prices where possible. Health related quality of life (HRQoL) was measured at baseline and 18 months using EQ-5D. Cost-effectiveness was assessed by comparing incremental costs and marginal benefits. Sensitivity analysis was undertaken.

## Results

**Figure 2:**
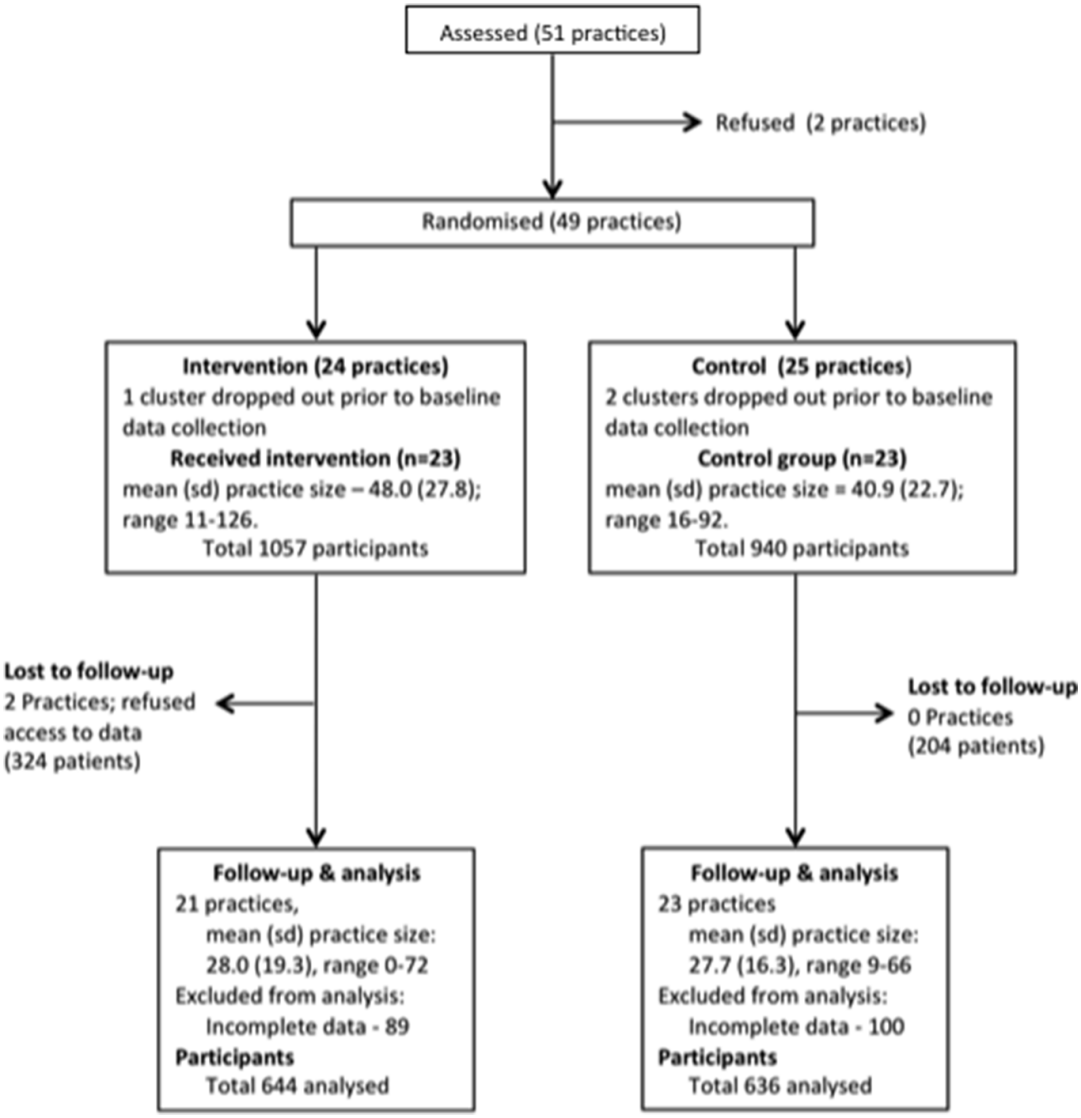
Consort flow diagram of GP Practices and patients. Consort diagram of practices and patients recruited, numbers followed up and included in analysis.

Initially 51 general practices were recruited to the study, but two dropped out prior to randomisation. 49 general practices were randomised, 24 to the intervention (a total of 1057 patients) and 25 to the control arm (a total of 940 patients). A further three practices withdrew prior to study commencement leaving 46 practices randomised, 23 to the intervention and 23 to the control arm. There were 11, 13 and 22 practices in PCT 1, PCT 2 and PCT 3 respectively. The CONSORT flow diagram of GP practices and participants through the study is shown in Figure 2.

### Attendance at ICCD

In PCTs 1 and 2, 145 (out of 431, 34%) and 35 (out of 240, 15%) trial participants were referred and attended the ICCDs, respectively. In PCT 3, the ICCDs were open to receive referrals to all the patients from the intervention practices and not just the trial patients. Therefore, the attendance of the trial patients in this PCT was estimated. In total, 101 patients attended ICCDs in PCT 3. As the proportion of the trial participation from the intervention practices in PCT 3 was 19%, we estimated 19 of the 101 patients attended the ICCDs were trial patients (out of 386, 5%).

### Baseline Data

Intervention and control participants were broadly similar at baseline with respect to gender, smoking status, co-morbidities, achievement of blood pressure, HbA1c and cholesterol targets and mean values of these variables (Table 1). There was a difference between groups with respect to combined control (primary outcome), with 11.2% of intervention vs. 8.7% of control patients achieving this. (P = 0.09, unadjusted chi-squared test). There were differences between PCTs in achievement of combined control at baseline; 7.2%, 9.4% and 12.6% in PCTs 1, 2 and 3 respectively.

**Table 1:** Baseline characteristics.

Variable	Control (n = 940)	Intervention (n = 1057)	p-value
	N (%)	N (%)	
PCT 1	242 (25.7)	431 (40.8)	<0.001
PCT 2	225 (23.9)	240 (22.7)	0.551
PCT 3	473 (50.3)	386 (36.5)	<0.001
Male	543 (58.1)	613 (58.4)	0.954
Smoking	118 (12.7)	116 (11.1)	0.305
**Comorbidity**			
Hypertension	505 (55.6)	612 (59.1)	0.067
IHD	161 (17.7)	149 (14.4)	0.071
CVD	35 (3.85)	28 (2.72)	0.214
Heart failure	25 (2.75)	35 (3.38)	0.471
PVD	10 (1.10)	15 (1.45)	0.609
Renal failure	24 (2.63)	24 (2.31)	0.791
**Ethnicity**			
White	614 (65.3)	554 (52.4)	<0.001
Asian	257 (27.3)	405 (38.3)	<0.001
Black	32 (3.40)	55 (5.20)	0.063
Other	37 (3.94)	43 (4.07)	0.972
**Baseline assessment of outcome measures**			
Primary outcome (combined control)	81 (8.74)	116 (11.2)	0.09
Controlled HbA1c (< = 7.0%/53 mmol/mol)	497 (53.9)	536 (51.7)	0.357
Controlled blood pressure (<140/80 mmHg)	304 (32.8)	398 (38.3)	<0.001
Controlled cholesterol (<154 mg/dl/4 mmol/l)	442 (48.2)	519 (50.2)	0.377
**Individual factors (mean/sd)**			
HbA1c	7.26 (1.24)	7.34 (1.40)	0.183
Systolic BP	137.4 (17.5)	136.9 (17.3)	0.525
Diastolic BP	80.8 (10.5)	79.5 (10.7)	0.007
Total Cholesterol	4.05 (1.04)	4.03 (1.13)	0.686

### Follow up Data

Two intervention practices refused access for follow up assessment and including these, a total of 528 patients were lost to follow up (intervention–324; control–204). A further 189 patients were excluded from analysis (intervention–89; control–100) due to incomplete data. Thus, data from 68% of the control group (636/940) and 61% of the 644/1057 intervention group were available for final analyses (Figure 2). There was some variation in follow up rates by PCT: 383/673 (57%) in PCT 1, 285/465 (61%) in PCT 2, and 612/859 (71%) in PCT 3. Table S1 shows percentages and numbers of patients with missing data for baseline, and similarly for follow-up. These missing values are for the primary outcome variable, individual secondary outcome variables, each of the covariates used in the primary analysis and before LOCF was applied. Baseline characteristics of those patients who were included in the analysis are presented in Table 2. While the descriptive characteristics were similar, there was no significant difference between intervention and control participants in the number of people who had ‘controlled blood pressure’ (Table 1 and Table 2).

**Table 2:** Baseline characteristics for patients included in analysis.

Variable	Control (n = 636)	Intervention (n = 591)	p-value
	N (%)	N (%)	
PCT 1	164 (25.8%)	166 (28.1%)	0.399
PCT 2	339 (53.3%)	237 (40.1%)	<0.001
PCT 3	133 (20.9%)	152 (25.7%)	0.054
Male	370 (58.2%)	347 (58.7%)	0.894
Smoking	77 (12.1%)	66 (11.2%)	0.672
**Comorbidity**			
Hypertension	341 (53.6%)	335 (56.7%)	0.307
IHD	115 (14.9%)	95 (16.1%)	0.392
CVD	22 (3.46%)	15 (2.54%)	0.438
Heart failure	15 (2.36%)	17 (2.88%)	0.697
PVD	7 (1.10%)	9 (1.52%)	0.690
Renal failure	13 (2.04%)	12 (2.03%)	1.000
**Ethnicity**			
White	365 (57.4%)	271 (45.9%)	<0.001
Asian	98 (15.4%)	202 (34.2%)	<0.001
Black	20 (3.14%)	33 (5.58%)	0.050
Other	20 (3.14%)	22 (3.72%)	0.690
**Baseline assessment of outcome measures**			
Primary outcome (combined control)	61 (9.59%)	76 (12.9%)	0.084
Controlled HbA1c (< = 7.0%/53 mmol/mol)	347 (54.6%)	326 (55.2%)	0.878
Controlled blood pressure (<140/80 mmHg)	354 (55.7%)	324 (54.8%)	0.812
Controlled cholesterol (<154 mg/dl/4 mmol/l)	305 (48.0%)	308 (52.1%)	0.162
**Individual factors (mean/sd)**			
HbA1c	7.22 (1.24)	7.18 (1.23)	0.470
Systolic BP	137.5 (17.3)	137.0 (18.0)	0.528
Diastolic BP	80.6 (10.0)	79.3 (10.7)	0.005
Total Cholesterol	4.05 (1.04)	3.99 (1.18)	0.231

### Primary Outcome

At follow up 14.3% of patients in the intervention group vs. 9.3% in the control group achieved combined control (Table 3). Percentage achievement (intervention vs. control) was 13.7 vs. 11.0, 10.5 vs. 11.3 and 14.2 vs. 7.7 in PCTs 1, 2 and 3 respectively. Of the 1280 patients observed at both baseline and follow-up, 33 were excluded from the regression analysis owing to missing data values for one or more covariates. The odds ratio for achievement of combined control for intervention vs. controls was 1.56 (95%CI 0.93, 2.49) (Table 4). Table 5 shows the full regression model showing all covariates.

**Table 3:** Follow-up outcome measures (n = 1280).

Variable	Control (n = 636)	Intervention (n = 644)	p-value
	N (%)	N (%)	
Primary outcome (combined control)	59 (9.3)	92 (14.3)	0.007
**Secondary outcome measures: achieved targets**			
HbA1c (< = 7.0%/53 mmol/mol)	325 *(51.1)*	370 *(57.5)*	0.026
Blood pressure (<140/80 mmHg)	203 *(32.0)*	256 *(39.8)*	0.004
Cholesterol (<154 mg/dl/4 mmol/l)	351 *(55.2)*	397 *(61.8)*	0.022
**Individual factors (mean/sd)**			
HbA1c	7.28 (1.36)	7.17(1.37)	0.150
Systolic Blood Pressure	138.0 (17.9)	136.9 (17.9)	0.272
Diastolic Blood Pressure	80.5 (10.2)	79.1 (10.7)	0.017
Total cholesterol	3.90 (1.11)	3.79 (1.01)	0.064

**Table 4:** Odds Ratios for primary and secondary outcomes.

	Odds ratio	95% confidence interval
Primary outcome (combined control)	1.56	0.983, 2.49
Secondary outcomes		
HbA1c control (< = 7.0%/53 mmol/mol)	1.45	1.07, 1.96
Blood Pressure control (<140/80 mm Hg)	1.23	0.88, 1.73
Total Cholesterol (<154 mg/dl/<4 mmol/L)	1.48	1.08, 2.03

**Table 5:** Odds ratio of achieving primary outcome with full regression model.

Fixed effects	Estimate	SE	p-value	Odds ratio	95% CI
Intervention arm	0.447	0.237	0.059	1.56	0.983, 2.49
Controlled at baseline	2.26	0.216	<2×10^−16^	9.61	6.29, 14.7
Male	−0.113	0.196	0.564	0.893	0.609, 1.31
Age at baseline	0.002	0.010	0.869	1.000	0.983, 1.02
PCT Site 2	−0.202	0.399	0.613	0.817	0.374, 1.79
PCT Site 3	−0.420	0.286	0.143	0.657	0.375, 1.15
Deprivation index	0.003	0.008	0.710	1.000	0.988, 1.02
Smoker	−0.276	0.373	0.460	0.759	0.365, 1.58
Hypertension	−0.420	0.211	0.046	0.657	0.435, 0.99
IHD	−0.044	0.250	0.861	0.957	0.586, 1.56
Cerebrovascular disease	0.427	0.370	0.248	1.56	0.743, 3.17
Heart failure	−1.87	1.06	0.077	0.154	0.019, 1.22
Peripheral vascular disease	−1.22	0.774	0.115	0.295	0.065, 1.35
Renal failure	−0.130	0.498	0.794	0.878	0.331, 2.33
Asian	−0.051	0.258	0.842	0.950	0.573, 1.57
Black	−0.054	0.548	0.922	0.947	0.324, 2.77
Other	0.082	0.472	0.862	1.09	0.430, 2.73

### Secondary Outcomes

Intervention group patients were more likely to achieve control of HbA1c and total cholesterol at follow-up (Table 4). Mean values of individual components of combined control are shown in Table 3. For all components, after adjustment for covariates, differences in change were small but favoured the intervention group and for cholesterol the difference was statistically significant (p = 0.014). Approximate intra-class correlation coefficients were as follows: HbA1c: 0.036, systolic blood pressure: 0.037, diastolic blood pressure: 0.051, cholesterol: 0.061.

### Economic Results

The unit cost per ICCD consultation is shown in Table 6. The mean cost of an ICCD consultation was £102.18 (range £74.01–£154.75). The overall cost of ICCD visits per patient referred to the service was £337.36 (range £253.74–£773.74).

**Table 6:** Cost of patient consultation at ICCDs.

PCT	Total costs	Total consultations	Patients attending ICCD	Average cost per consultation	Average cost per patient attending ICCD
1	£43,553	442	145	£98.54	£300.37
2	£8,881	120	35	£74.01	£253.74
3 (trial patients only)	£14,701*	95	19	£154.75	£773.74
Total	£67,135	657	199	£102.18	£337.36

*During the trial period, there were 500 consultations, each costed at £154.75. This gives a total cost of £77,375, of which 19% (£14,701) was attributed to trial patients.

Other direct healthcare costs for patients in the two trial groups are shown in Table 7. These figures are based on 1322 patients reporting resource use. Analysis of community consultations indicates that the mean primary care (GP and practice nurse visits) cost per patient was slightly higher in the ICCD group (£37.25 vs. £31.19, p = 0.051); community clinic costs were significantly higher (£1.46 vs. £ 0.49, p = 0.025); differences in other costs were not significant. Overall, total consultation costs (including ICCD visits) were significantly higher in the intervention group; £137.70 (SE = £5.53) vs. £76.82 (SE = £6.25), p<0.001). The total cost of care per patient (including diagnostic tests and hospital inpatient stay) was higher in the intervention group (£351.68 vs. £238.52), but this difference did not reach statistical significance (p = 0.247).

**Table 7:** Direct healthcare costs by resource category.

Resource item	Intervention (n = 665)		Control (n = 657)		p-value
	Mean	SE	Mean	SE	
Intermediate care clinic for diabetes	60.18				
**Cost of consultations**					
Primary care doctor and nurse costs	37.25	2.335	31.19	2.044	0.051
Community clinic staff	1.46	0.381	0.49	0.201	0.025
Hospital doctor and nurse costs	26.13	3.876	32.03	5.272	0.366
AE staff	1.02	0.525	0.59	0.295	0.476
Optometrist, podiatrist and dietician	11.65	1.047	12.51	0.907	0.534
**Sub total**	**137.70**	**5.53**	**76.82**	**6.25**	**<0.001**
**Cost of care**					
Diabetes tests	58.27	2.27	62.74	2.63	0.199
Hospital inpatient costs	155.71	75.60	98.96	58.67	0.554
**Total costs**	**351.68**	**76.51**	**238.52**	**60.70**	**0.247**

There was no significant difference in HRQoL between patient groups at baseline (Table 8). An incremental cost-effectiveness ratio (ICER) of +£7,778 per QALY gained, following 1,000 replicated bootstraps, indicated that ICCD was marginally more expensive at producing health gain (EQ-5D) for patients.

**Table 8:** Health related quality of life by EQ-5D.

	Intervention (n = 289)	Control (n = 262)	p-value
	Mean (SE)	Mean (SE)	
Baseline	0.69 (0.02)	0.7 (0.02)	0.575
Follow-up	0.7 (0.02)	0.7 (0.02)	0.982

## Discussion

Our results show that providing practices with access to an ICCD service led to an increase in the proportion of patients achieving targets for control as assessed by our primary outcome measure (control of HbA1c, BP and cholesterol), although this just failed to reach statistical significance. With adjustment for the effects of clustering and potential confounding, the odds ratio for achieving the combined control was 1.56 (95% CI 0.98, 2.49). Results from the individual components of the outcome measure showed the intervention had a significant effect on achieving control of HbA1c and cholesterol, with less impact on blood pressure. The effect of the intervention on mean values of HbA1c, BP and cholesterol was modest. This achieved statistical significance only for cholesterol, but the mean decrease of 0.20 mmol/l in the intervention group compared with a decrease of 0.15 mmol/l in the control group is unlikely to be of clinical importance. However this is against a background of good levels of control of these factors in both groups at baseline, and other studies of service interventions have also shown only modest effects [22], [25].

Taken together, these findings suggest that the intervention was beneficial in effecting small changes in risk factors that enabled patients to cross the threshold between adequate and inadequate control. The proportion of trial participants attending ICCD varied across PCTs, perhaps reflecting differences in “case finding”. In PCTs 1 and 2, an active “case finding” approach was used in which members of the ICCD team searched GP records to identify those with suboptimal risk factor control who may benefit from referral. Although the ICCD service in PCT 3 visited practices to promote the clinics, it relied on primary care practitioners identifying suitable patients for referral. As well as the services being newly established, their introduction in the context of a trial could also have reduced uptake. The small changes in risk factor control may reflect low referral to ICCDs, which could have been improved by more active case management. ICCDs are one way to provide such an enhanced case management service in the community with specialist input. Integrated case management through “case finding” coupled with intensive intervention within existing primary care services and settings might be equally effective.

The remit of all ICCD services included education and up-skilling of primary care practitioners, and so it is likely that their impact extended beyond improving control in patients referred, for example by offering informal advice about patients without an actual referral. Other studies have identified the key problem of clinical inertia in primary care (a reluctance to step up treatment) as a barrier to improving diabetes care [26], [27]. It is likely that the ICCD service tackled this problem, both through direct work with patients and practice education. In addition, our qualitative study, to be reported separately, suggested both patients and primary care practitioners welcomed such enhanced service in the community, a finding consistent with other studies [28], [29].

Case management and workforce changes have both been identified recently as key components of effective diabetes care interventions [6]. Whilst the ICCDs are not explicitly constructed around these principles, the proactive “case finding” approach used in 2 and use of multi disciplinary teams in all 3 PCTs both represent first steps towards such strategies. Although this study was based in and focused only on the UK, the ICCDs might present a unique opportunity for any purchaser-provider model throughout the world. Indeed, if set up specific to the needs, such integrated, multi-disciplinary care teams might be more effective in other health care systems.

The economic analysis suggested that ICCD is cost neutral; increasing costs in primary care but having no significant effect on overall total cost per practice patient. However, this finding must be interpreted with caution because of the skewed nature of secondary care costs, the major cost driver. Sensitivity analysis indicated that the overall cost of diabetes care is highly dependent upon the inpatient costs of a small number of relatively expensive patients. During the trial existing facilities and staff were used. Additional start-up costs would be required if a new service were commissioned.

### Strengths and Weaknesses

The study was an ambitious attempt to evaluate an innovation in service provision that was being widely adopted, but for which there was no strong evidence of clinical or cost effectiveness. It is the only public-funded, randomised trial to-date, which succeeded in recruiting a large number of practices. These practices agreed to be randomised, and worked closely with three ICCD services during the trial period. It has provided the most robust evidence to-date on the effectiveness of this intervention. The main limitations of the trial were the low number of participants and higher than expected loss to follow up; although in a cluster randomised trial a reduction in size of cluster has less effect on power than a reduction of clusters, the study had less power than planned. The low participation rate as well as the variation in baseline characteristics between the groups may have introduced selection bias. In planning the study, we anticipated collection of follow-up data for around 70% of baseline participants. We achieved follow-up for 64% of patients, but with a significant excess of control compared to intervention patients, contributed to by two intervention practices dropping out of follow-up data collection. This is a known danger of cluster randomised controlled trials where practices (clusters) act as gatekeepers to patients and may decide to withdraw co-operation for pragmatic reasons, thus removing groups rather than individual participants who may be willing to continue.

## Conclusions

Our findings support the consideration of ICCD in the range of services commissioned to provide care for people with diabetes. This is especially relevant in the UK with the current change in NHS commissioning, with an emphasis on “personalised, integrated community based care”. We have also shown the importance of working closely with local practices to promote the service and that without a “case finding” approach such services may be under-utilised. Such integrated diabetes service in a “hub and spoke” model between primary and specialist services could utilise the specialist expertise economically and provide opportunity for regular “up skilling” the knowledge of primary care practitioners. This model can be easily implemented across the world and we speculate that a structured, pre-defined and proactive “case finding” approach can improve the quality of care, reduce cardiovascular mortality without increasing the overall cost.

## Supporting Information

Table S1 shows percentages and numbers of patients with missing data for baseline and for follow-up.
(DOCX)

Checklist S1 CONSORT checklist.
(DOC)

Protocol S1 Trial protocol.
(DOC)
