# Supplementary material for: Evaluation of the Clinical and Cost Effectiveness of Intermediate Care Clinics for Diabetes (ICCD): A Multicentre Cluster Randomised Controlled Trial
Source: PLoS One. 2014 Apr 15;9(4):e93964. doi: 10.1371/journal.pone.0093964 (PMC3988031; doi:10.1371/journal.pone.0093964)
Supplement: Table S1 — shows percentages and numbers of patients with missing data for baseline and for follow-up. (DOCX) [file pone.0093964.s001.docx]

**Table S1: Percentage use of LOCF.**

|  | Control | | | | Intervention | | | |
| --- | --- | --- | --- | --- | --- | --- | --- | --- |
|  | HbA1C | BP-S | BP-D | Lipids | HbA1C | BP-S | BP-D | Lipids |
| Overall | 5.97% | 24.5% | 24.7% | 15.1% | 7.92% | 17.2% | 17.2% | 11.0% |
| Age<55 | 6.56% | 12.3% | 21.3% | 15.6% | 6.67% | 21.7% | 21.7% | 7.50% |
| 55<=Age<=64 | 6.74% | 21.8% | 24.9% | 3.63% | 6.67% | 23.9% | 8.89% | 7.22% |
| 65<=Age<=72 | 15.9% | 14.4% | 23.4% | 13.8% | 7.07% | 23.4% | 15.8% | 14.1% |
| Age>72 | 4.93% | 26.9% | 29.6% | 17.6% | 11.9% | 29.1% | 24.5% | 15.2% |
| Male | 6.54% | 16.6% | 22.1% | 14.2% | 8.58% | 22.0% | 16.6% | 11.5% |
| Female | 5.34% | 18.7% | 29.0% | 16.8% | 7.17% | 28.3% | 18.5% | 10.6% |
| Ethnicity 1 | 5.90% | 26.8% | 26.8% | 15.7% | 10.3% | 20.0% | 20.0% | 14.3% |
| Ethnicity 2 | 0% | 33.3% | 3.33% | 0% | 0% | 14.3% | 14.3% | 0% |
| Ethnicity 3 | 8.00% | 22.0% | 22.7% | 18.7% | 5.03% | 17.1% | 17.1% | 6.54% |
| Ethnicity 4 | 0% | 6.25% | 6.25% | 0% | 0% | 0% | 0% | 0% |
| Ethnicity 5 | 0% | 0% | 0% | 0% | 0% | 0% | 0% | 14.3% |
| Ethnicity 6 | 0% | 9.09% | 9.09% | 9.09% | 0% | 20.0% | 20.0% | 0% |
| Ethnicity 7 | 0% | 50.0% | 50.0% | 0% | 0% | 0% | 0% | 0% |
| Ethnicity 8 | 8.33% | 37.5% | 37.5% | 8.33% | 15.2% | 6.06% | 5.06% | 18.2% |
