# Supplementary material for: Evaluation of the Clinical and Cost Effectiveness of Intermediate Care Clinics for Diabetes (ICCD): A Multicentre Cluster Randomised Controlled Trial
Source: PLoS One. 2014 Apr 15;9(4):e93964. doi: 10.1371/journal.pone.0093964 (PMC3988031; doi:10.1371/journal.pone.0093964)
Supplement: Protocol S1 — Trial protocol. (DOC) [file pone.0093964.s003.doc]

Evaluation of **I**ntermediate **C**are **C**linics for **D**iabetes (ICCD)

Version 3, 27/06/2006

**University of Leicester**

Andrew Wilson, Reader in General Practice

Kamlesh Khunti, Clinical Senior Lecturer

Melanie Davies, Professor of Diabetic Medicine

Richard Baker, Professor of Quality in Health Care

**University of Warwick**

Sudhesh Kumar, Professor of Medicine (Diabetes and Metabolism)

Hillary Hearnshaw, Reader in Primary Care

Jeremy Dale, Professor of Primary Care

Ala Szczepura, Professor of Health Services Research

Neil Raymond, Lecturer in Epidemiology

Anil Gumber, Senior Research Fellow

Nigel Stallard, Professor of Medical Statistics

**CONTENTS**

|  | Page |
| --- | --- |
| Summary | 3 |
| Aims, Objectives, Background | 4 |
| Local context, Plan of investigation | 5 |
| Methods | 6 |
| Provisional Timetable | 11 |
| Research Funding | 14 |
| References | 15 |
| Appendix 1: Establishing capacity in intermediate care clinics | 17 |
| Appendix 2: NHS Support costs | 18 |
| Appendix 3: Patient information sheet | 19 |
| Appendix 4: Patient consent form | 22 |
| Appendix 5: GP invitation letter | 23 |
| Appendix 6: Patient assessment pack | 24 |
|  |  |

Evaluation of **I**ntermediate **C**are **C**linics for **D**iabetes (ICCD)

**SUMMARY**

## Intermediate care clinics (ICC) are suggested as one method of improving care for people with type 2 diabetes, but their effectiveness and cost effectiveness is not known. Their aim is to provide a multidisciplinary community based service to support general practices in achieving good control of their patients. Four local PCTs (Rugby, North Warwickshire, Eastern Leicester and Leicester City West) have agreed to establish intermediate care clinics as part of a cluster randomised trial of their effectiveness.

## All patients with type 2 diabetes in participating practices will be invited to take part. Those that agree will attend a baseline assessment by a study nurse. This will include measurement of HbA1C, body mass index, waist circumference,blood pressure and lipids, as well as questionnaires about quality of life, satisfaction with current services and continuity of care. Practices will then be randomised to either usual care or intervention arms, the latter having access to the new clinics. Participating patients will be asked to attend a follow up assessment 18-24 months after the baseline assessments, when the same measurements will be repeated. We expect about 30% of patients in the intervention arm will have been referred to the ICC.

## The primary outcome will be the percentage of patients achieving adequate control of HbA1, blood pressure and cholesterol, comparing all participants in the intervention and control arms, whether or not those in the intervention arm attended ICC. The study is powered to detect a 10% difference in this outcome, and will include 51 practices and 5100 patients. We will also examine the effect of the intervention on process measures, such as the number of contacts in hospital and general practice. In the intervention arm we will document in detail the inputs provided by ICC, so that if successful the model can be adopted elsewhere.

The economic evaluation will be undertaken from a societal perspective. A costing study will measure intervention and treatment costs in both groups. A comparative assessment of the marginal costs and outcomes of the intervention will be undertaken, to include cost-effectiveness ratios and cost-utility analysis. All findings will be subject to sensitivity analysis.

Additionally we will explore the views of patients in the intervention arm who attended ICC using semi-structures interviews to identify positive and negative aspects of the patients’ experiences. We will also hold focus groups to explore views of professional stakeholders.

Evaluation of **I**ntermediate **C**are **C**linics for **D**iabetes (ICCD)

**AIM**

To evaluate the effectiveness and cost effectiveness of community based intermediate care clinics (ICC) in the management of type 2 diabetes.

**OBJECTIVES**

1 To compare the following in patients with type 2 diabetes registered with practices that have access to ICC with those that have access only to usual care

- Control of cardiovascular risk factors including glycaemic control, blood pressure and dyslipidaemia
- Quality of life
- Satisfaction with services and continuity of care
- Referral patterns and non attendance rates
- Annual cost per patient with diabetes

2 To assess the cost effectiveness of the intervention

3 To explore the views users and service providers have of ICC

**BACKGROUND**

There is excellent evidence from randomised controlled trials that intensive management of type 2 diabetes reduces complications such as retinopathy, nephropathy and neuropathy, as well as reducing the risk of cardiovascular disease.1 Benefits are seen from improved glycaemic control2, lower blood pressure3 and management of lipids.4 Patients with better glycaemic control also report better functional status and wellbeing.5

We also know in principle how the benefits seen in explanatory trials can be realised in practice. A Cochrane review found that structured recall, patient education, and support from specialist nurses led to better outcomes,6 and a trial in Danish general practice found that intensive patient focussed management led to significant risk reduction.7 Economic modelling has suggested that intensive control of risk factors is also likely to be cost effective in the UK.8

However, configuring services so that all people with diabetes can benefit from good quality, target driven and patient focused care is a major challenge to the NHS, especially given the increased prevalence of the condition. In recent years there has been a shift in place of care from hospital to general practice9 and currently about 70-80% of people with type 2 diabetes are managed entirely in primary care, with evidence of deficiencies in provision of care and significant variations between general practices.10 11 Practices in areas of high deprivation and mix of ethnicity are less likely to achieve adequate levels of control. Although the new contract for general practices has set targets for the control of diabetes, concerns about whether primary care can continue to cope without additional support are still voiced.12

One method of supporting the primary care management of diabetes is intermediate care clinics (ICC), many of which are being established nationally. (<http://www.diabetes.audit-commission.gov.uk/casestudies/improving2.htm>) These aim to deliver high quality care by a multidisciplinary locality based team, with opportunities for developing professional expertise in the community. A common model is for medical care to be provided by GPs with a special interest (GPwSI)13 working with community based specialist teams. There have been no formal evaluations of ICC on patient outcomes. A descriptive evaluation found intermediate clinics were popular with patients and practitioners and reduced outpatient attendances by 25%.^14^ However, there could be a trade off between the higher levels of technical expertise available in ICC and continuity (which has been shown to be related to glycaemic control^15^) provided by practice based approaches.

**LOCAL CONTEXT**

Providing good quality care for people with diabetes is a particular challenge for the health services in Leicester and Warwickshire given the high prevalence of the condition, particularly in minority ethnic groups. The prevalence of diabetes in Eastern Leicester PCT is in the top 10 nationally.^16^

Participating PCT and the number of practices they contain is listed below

Eastern Leicester: 35 practices (45 surgeries)

Leicester City West: 26 practices (31 surgeries)

North Warwickshire: 25 practices (39 surgeries)

Rugby: 12 practices (16 surgeries)

TOTAL 98 practices (131 surgeries) [data accessed 22 Feb 2006]

The ICCs will be community based in all participating PCTs. Their aim is to support primary care, particularly smaller practices that have the most difficulty achieving good control. They will comprise a multidisciplinary team, including specialist nurses who will place an emphasis on education and self management. Medical care will be provided by a GPwSI and/or community based diabetologist. The ICC will work closely with hospital based specialist teams and community services including podiatry and dietetics. Team members will work to local guidelines, adapted from national evidence based guidelines ([http://www.leicestershirediabetes.org.uk](http://www.leicestershirediabetes.org.uk/)) Guidelines for referral to the ICCs will be common across all sites, and will include people with poorly controlled type 2 diabetes and those with poorly controlled cardiovascular risk factors.

Patients will be managed by the ICC team until maximal control is achieved and then referred back to primary care. Patients with type 2 diabetes will no longer be referred to hospital directly. At all sites the planned capacity is for 20% of patients with type 2 diabetes to attend the ICC per annum, assuming that on average patients will be seen four times in the ICC before returning to primary care.

**PLAN OF INVESTIGATION:**

Practices recruited to the study will be randomised to the intervention or control arm. Practices in the intervention arm will have access to ICC. Those randomised to usual care will be issued with local guidelines and continue to manage their patients, including hospital referral, in the usual way. All patients with type 2 diabetes in participating practices will be invited to take part and asked to attend for baseline assessment and follow up assessment at the end of the 18 month intervention period. The primary outcome is the percentage achieving control of HbA1C, cholesterol and blood pressure. At the end of the trial focus groups will be used in the intervention arm to elicit user and stakeholder views. The trial outline and expected numbers are shown in the flowchart below.

Number of practices in participating PCTs **98**

Recruitment to study **51**

INTERVENTION

CONTROL

Randomisation by size 25-26 25-26

and PCT

Patients with T2DM 3825 3825

Patients consenting (70%)

Initial assessment 2550 2550

Usual care

ICC

Usual care

18month intervention period 70% 30%

(1785) (765)

Final assessment

**1250 535**

**1785**

(70% follow-up)

**INTENTION TO TREAT ANALYSIS**

**METHODS**

*Practice recruitment*

All practices in participating PCTs will be invited to express interest in taking part. Experience from a similar trial of community cardiology clinics in Leicester suggests that over 80% of practices will agree, as it gives them an even chance of accessing an enhanced service for their patients.18

Practices that express interest will be asked to provide data on current levels of control of their patients with type 2 diabetes (data on % patients with HbA1C <7.5, BP145/85 or less and cholesterol 5mmol/l or less is routinely collected as part of the GP contract19) and those with the highest proportions of poorly controlled patients will be invited to join the study, with the aim of recruiting a total of 51 practices (52% of all eligible practices).

*Patient recruitment and retention*

All patients with type 2 diabetes on participating practices’ registers will the invited to take part in the study, including those currently attending hospital. Exclusion criteria are severe mental illness, severe cognitive impairment and receiving terminal care. Patients will be approached by a letter from their GP enclosing a study information sheet and reply slip. Those who express interest will be contacted by research team who will provide further information and, if the patient is agreeable, arrange a date for the baseline assessment, at which time written consent will be obtained by a research nurse. In a similar study in Denmark 94% consented 7, but assuming a lower consent rate of about 70%, as seen in UKADS26 we will expect an average sized participating practice with 150 patients with diabetes to recruit 100 of them. We will send patients a newsletter about the study at the midpoint to improve follow up rates. Those not responding to requests for follow up attendance will be reminded using letters, telephone, email and texting.

*Randomisation of practices*

After the baseline assessments practices will be randomised to intervention or control groups. This sequence will avoid the risk of patients in intervention practices being more likely to agree to take part. Randomisation of practices will be stratified by PCT and practice size, to achieve similar numbers in each arm of the trial, and done by the Leicester clinical trial support unit.

*Outcome measures:*

The primary outcome These targets, although not reflecting optimal control, are those set in the GP contract19, and if achieved would represent significant health gain. We want to be able to detect a 10% difference in this level of control in the intervention arm, whether or not they attended ICC, compared with the usual care group. (We expect approximately 30% of those eligible to attend ICC over the 18 month study period) Audit data suggest that in participating practices about 15% of patients will have this level of control at baseline (Leic Primary Care Audit Group, personal communication) and we expect an increase of no more than 5% in the control arm.

Secondary outcomes are % reaching target for individual risk factors (blood pressure, HbA1c and cholesterol) and other biomedical markers, including body mass index and waist circumference, as well as use of medication, reported smoking, costs, quality of life, emotional adjustment, patient satisfaction and views on continuity

*Process measures*

We will measure the number of referrals to and attendances at ICC in the intervention arm and to hospital in the control arm, and examine practice variation. We will also note the number of GP and practice nurse consultations, and total hospital attendances both inpatient and outpatient. For patients who are referred to ICC we will note number of contacts and with which professional, as well as any group or educational intervention. These data will be obtained from practice records at the time of the follow up assessment.

*Patient assessments*

A study nurse will conduct these assessments on practice premises or at home if the patient is housebound. Baseline assessments are necessary as GP data is non standardised and will be up to a year old. It will not be feasible for study nurses to be blinded to allocation, but outcome measures are all objective; an automated sphygmomanometer will be used, and the bloods analysed in a hospital laboratory. As assessments will take part throughout the day, obtaining fasting blood samples is not feasible.

Biomedical markers will consist of blood pressure, blood lipids, HbA1c, BMI, waist circumference, proteinuria and self reported smoking. Quality of life will be assessed using EQ-5D21 and the short version of World Health Organisation’s Quality of Life. We will also assess satisfaction with services (DTSQ22) emotional functioning (PAID23) and continuity of care using a questionnaire recently developed by Gulliford in an SDO funded study. (Annals of Family Medicine, in press)

All these questionnaires will be used at both assessments. Additionally the baseline assessment will include demographic data, and the follow up assessment questions about health related costs borne by the patient. Use of NHS resources will be assessed from the GP record at the follow up assessment. Data extracted will include number of GP and practice nurse contacts, hospital attendances as in patient or outpatient, and prescribed medication and investigations. In the intervention group contacts with the ICC will also be extracted from the GP and ICC records. The GP records of patients will be flagged electronically to encourage reporting of death or change of GP. In-patient and out-patient attendances will also be assessed by interrogating hospital datasets using participants’ NHS numbers. Data extractors will be suitable trained, and checks made on inter-rater reliability.

At baseline assessment the GP will be advised only of any findings that put the patient at immediate risk. GPs will be informed of all results at follow up assessment, This will help in the recruitment of practices, as it will be of value to those in the control as well as intervention arm.

*Patients whose preferred language is not English*

We will use translated versions of questionnaires if available (for example the EQ5D) When translated versions of questionnaires are not available we will suggest that the English version is completed with the help of a family member of friend, and will explore whether exclusion of these affects results. All trial literature will be translated, using resources available at Warwick University.

*Timetable for participating practices*

It is expected that the launch of the trial will be phased across participating PCTs. The sequence of events is shown in the table below, combining N Warks and Rugby PCTs. In each of the three centres practice and recruitment will take place over 6 weeks, followed by patient recruitment. Baseline assessments will then take place over 3 months, following which practices will be randomised and the clinic launched. The clinic will then run for 18 months, after which follow up assessments will be undertaken. This ensures that all patients in the intervention arm have 18 months ‘exposure’ to the ICC but means that follow up assessments will be undertaken 21-22 months after recruitment, not at 18 months as stated in the original protocol.

|  | no weeks | cumulative weeks |
| --- | --- | --- |
| Write to practices | 2 |  |
| Visit to discuss /confirm participation (n=15-20) | 4 | 6 |
| Patient recruitment | 4 | 10 |
| Baseline assessments of patients/qof data | 13 | 23 |
| Randomisation of practices and notification | 1 | 24 |
| **Launch of clinic** | 1 | 25 |
| Clinic runs for 18 months (SFS stops) | 78 | 103 |
| Follow up assessments | 13 | 116 |

*Power*

Estimates for % patients achieving control in the usual care group are taken from a recent UK survey.11 We have used HbA1c for our primary sample size calculation as this is the outcome variable for which there is the most robust information on intra-class correlation (ICC). To detect a difference of percentage well controlled (HbA1c <7.5%) from 50% in control group to 60% in intervention group (alpha = 0.05 Power = 0.8) not allowing for clustering requires a sample size of 408 subjects in each arm. The best estimate for the ICC for HBA1c is 0.047, in a study of newly diagnosed patients25 although smaller studies on established patients have produced similar estimates.26 Using an ICC of 0.047 and with 72 patients in each cluster, the design multiplier is 1+ (0.047x71)= 4.34, inflating the sample size to 1770 in each arm, a total of 3540. This sample would also be adequate to detect a 10% difference in cholesterol control (from 60% to 70%, ICC 0.05, 3421 needed) and blood pressure control (from 60% to 70%, ICC 0.03, 2353 needed) Estimates of ICC for blood pressure and cholesterol are taken from UKADS, a study of care provision for people of South Asian ethnicity with diabtes26 (Raymond, personal communication, unpublished data) Assuming the ICC for our combined primary outcome (adequate control of HbA1C, blood pressure and cholesterol) is 0.05 and achievement by 20% in the control arm and 30% in the intervention arm would need 2194 patients. In summary the trial is adequately powered to detect differences in control of the three individual outcomes, and well powered to detect differences in the combined outcome.

*Sample size/Recruitment*

An average practice of 6000 patients will have approximately 150 patients with type 2 diabetes. Of the average 100 patients recruited by each practice we expect between 70% and 90% of patients will attend follow up and complete the required questionnaires (allowing for patients moving away and drop-outs) A similar study achieved 75% follow up at 12 months,25 although UKADS26 successfully followed up 90% at one year, and a similar rate (88%) was achieved by RIGHT.(Raymond, personal communication) We have conservatively based our power calculations on 70% follow up.

The total number of practices in participating PCTs is 98. We aim to recruit 51 practices and 5100 patients, of whom 70% (3570) will be expected to attend follow up, thereby achieving our required sample size. If patient recruitment rates are lower than expected we will offer the study to additional practices.

*Analysis*

The main analysis will concentrate on the primary study outcome, proportions achieving all three targets; estimating OR with 95% confidence intervals for the intervention compared to control patient groups, adjusting for potential confounding variables and allowing for the effect of the cluster randomisation.27 The analyses will be on an intention to treat basis, with patients remaining in their allocated cluster even if follow-up data are not available. As some drop-outs and losses to follow-up are inevitable, sensitivity style analyses are planned; two types of missing data imputation will be used; first using previously recorded levels for biomarkers (last value forward - assuming no improvement or deterioration), and second using mean practice follow-up values. Data will also be analysed excluding all observations with missing values. Sample size has been inflated to allow for drop-outs, to allow a fully powered analysis based on per protocol allocation.

Further analyses will investigate secondary outcomes; achievement of individual targets for blood pressure, HbA1c and lipids, again adjusting for confounders and allowing for the cluster randomisation.

*Economic analysis*

The economic evaluation will be undertaken from a societal perspective (including patient costs). A costing study will measure intervention and treatment costs and differences in these costs between intervention and control groups. Short term cost estimates will be complemented by longer term cost consequences of diabetic complications. A comparative assessment of the marginal costs and outcomes of the intervention will be undertaken, to include cost-effectiveness ratios (based on HbA1c changes) and cost-utility analysis. The number of visits to ICC and hospital by patients and incurred health related costs in terms of travel expenses, time off work and other personal expenditure will be collected. The volume of resource use and associated costs in terms of visits to Consultants, GPwSI, specialist nurse, dietician, podiatrist and health care assistants will be estimated using unit cost approach. The EQ-5D instrument will be used to generate utility scores and the cost-utility analysis will provide an estimate of incremental cost of any benefit gained in terms of improved health status. The analyses will be undertaken both in summary form in terms of incremental cost per QALY, and also using a 'disaggregated' approach where the extra costs are presented alongside the outcome gains in terms of improvements in mobility, self-care, usual activity, pain/discomfort and mental well-being. In all these analyses, the uncertainties in the cost and outcomes data will be incorporated into a sensitivity analysis.

*Qualitative study*

In order to understand the reasons why the ICC might be deemed effective or ineffective, qualitative data concerning the stakeholders’ and participants’ views and experiences of the ICC will be collected, analysed and interpreted. Summary data from the trial results will be used to stimulate participants’ ideas about the factors which helped or hindered the implementation of the ICC and its impact on diabetes control. Semi-structured exploratory interviews will be undertaken with a sample of 20 patients who have experienced the intervention. This will be purposive sample, based on responses to the quantitative questionnaires administered at the end of the study. For patients requiring a carer, both the patient and carer will be interviewed. Where a patient does not speak English a translator will be engaged, recognising the limitations this may have on the interview. This will enable interviews to be conducted in Punjabi, Gujurati or Urdu. This resource will be available from the newly established Leicester Centre for Ethnic Health Research, and is already being used in the DESMOND trial. The interview will focus on the patient’s experience of the ICC, and will explore the positive and negative aspects, compared to their previous usual care. Wherever possible, interviews will be undertaken face to face. However, if necessary the interview may be undertaken by telephone.

In addition, focus groups of professional stakeholders will provide a forum for the discussion of issues related to the establishment and provision of the ICC. The groups will comprise practitioners who refer to the ICC, clinicians providing care at the ICC, hospital diabetologists and managers from the host PCTs. We anticipate holding 6 focus groups, if possible, with staff from more than one participating PCT, in each group. This will allow for sharing of views and experiences of different ICCs, thus widening the breadth of data gathered. The proximity of participating PCTs will enable this. Tapes will be transcribed and analysed using framework approach.^28^

Topic guides and will be developed following analysis of the quantitative data. These guides, as well as participant information sheets and consent forms will be approved by the ethics committee before interviews and focus groups take place.

**PROVISIONAL TIMETABLE**

The timetable shown below will be amended if PCT launch dates alter.

|  |  |  |
| --- | --- | --- |
|  |  |  |
| **STUDY BEGINS** | **start june 06** | Appointment of trial coordinator for 3y |
| **Piloting, preparation of materials** | **june-july** |  |
| pct 1 practice recruitment/randomisation | aug-sept 06 |  |
| pct 1 baseline assessments | oct -dec 06 | 4.5 nurses employed for 12m |
| **pct 1 clinic launch (NWARKS/RUGBY)** | **start january 07** |  |
| pct 2 practice recruitment/randomisation | nov-dec 07 |  |
| pct 2 baseline assessments | jan-march 07 |  |
| **pct 2 clinic launch (LCW PCT)** | **start april 07** |  |
| pct 3 practice recruitment/randomisation | feb-march |  |
| pct 3 baseline assessments | april-june |  |
| **pct 3 clinic launch (ELPCT)** | **start july 07** |  |
| pct 1 clinic closes for trial | end june 08 |  |
| pct 1 follow up assessments | july-sept 08 | 3 nurses employed for 12m |
| pct 2 clinic closes for trial | end sept 08 |  |
| pct 2 follow up assessments | oct -dec 08 |  |
| pct 3 clinic closes for trial | end dec 08 |  |
| pct 3 follow up assessments | jan- march 09 |  |
| Focus groups | jan-march 09 |  |
| **Final report** | **end May 09** |  |
| Interim reports | 6 monthly |  |
| Steering group | 6 monthly |  |
|  |  |  |

|  | **Year 1** | | | | | | | | | | | | **Year 2** | | | | | | | | | | | | **Year 3** | | | | | | | | | | | |
| --- | --- | --- | --- | --- | --- | --- | --- | --- | --- | --- | --- | --- | --- | --- | --- | --- | --- | --- | --- | --- | --- | --- | --- | --- | --- | --- | --- | --- | --- | --- | --- | --- | --- | --- | --- | --- |
|  | **June 06 - May 07** | | | | | | | | | | | | **June 07 - May 08** | | | | | | | | | | | | **June 08 - May 09** | | | | | | | | | | | |
|  | 1 | 2 | 3 | 4 | 5 | 6 | 7 | 8 | 9 | 10 | 11 | 12 | 1 | 2 | 3 | 4 | 5 | 6 | 7 | 8 | 9 | 10 | 11 | 12 | 1 | 2 | 3 | 4 | 5 | 6 | 7 | 8 | 9 | 10 | 11 | 12 |
|  | j | j | a | s | o | n | d | j | f | m | a | m | j | j | a | s | o | n | d | j | f | m | a | m | j | j | a | s | o | n | d | j | f | m | a | m |
| **Piloting preparation of materials** |  |  |  |  |  |  |  |  |  |  |  |  |  |  |  |  |  |  |  |  |  |  |  |  |  |  |  |  |  |  |  |  |  |  |  |  |
| **PCT1 Practice recruitment/randonisation** |  |  |  |  |  |  |  |  |  |  |  |  |  |  |  |  |  |  |  |  |  |  |  |  |  |  |  |  |  |  |  |  |  |  |  |  |
| **PCT1 Baseline assessment** |  |  |  |  |  |  |  |  |  |  |  |  |  |  |  |  |  |  |  |  |  |  |  |  |  |  |  |  |  |  |  |  |  |  |  |  |
| **PCT1 Clinic launch & finish** |  |  |  |  |  |  |  |  |  |  |  |  |  |  |  |  |  |  |  |  |  |  |  |  |  |  |  |  |  |  |  |  |  |  |  |  |
| **PCT2 Practice recruitment/randomisation** |  |  |  |  |  |  |  |  |  |  |  |  |  |  |  |  |  |  |  |  |  |  |  |  |  |  |  |  |  |  |  |  |  |  |  |  |
| **PCT2 Baseline assessment** |  |  |  |  |  |  |  |  |  |  |  |  |  |  |  |  |  |  |  |  |  |  |  |  |  |  |  |  |  |  |  |  |  |  |  |  |
| **PCT2 Clinic launch & finish** |  |  |  |  |  |  |  |  |  |  |  |  |  |  |  |  |  |  |  |  |  |  |  |  |  |  |  |  |  |  |  |  |  |  |  |  |
| **PCT3 Practice recruitment/randomisation** |  |  |  |  |  |  |  |  |  |  |  |  |  |  |  |  |  |  |  |  |  |  |  |  |  |  |  |  |  |  |  |  |  |  |  |  |
| **PCT3 Baseline assessment** |  |  |  |  |  |  |  |  |  |  |  |  |  |  |  |  |  |  |  |  |  |  |  |  |  |  |  |  |  |  |  |  |  |  |  |  |
| **PCT3 Clinic launch & finish** |  |  |  |  |  |  |  |  |  |  |  |  |  |  |  |  |  |  |  |  |  |  |  |  |  |  |  |  |  |  |  |  |  |  |  |  |
| **PCT1 Follow-up assessment** |  |  |  |  |  |  |  |  |  |  |  |  |  |  |  |  |  |  |  |  |  |  |  |  |  |  |  |  |  |  |  |  |  |  |  |  |
| **PCT2 Follow-up assessment** |  |  |  |  |  |  |  |  |  |  |  |  |  |  |  |  |  |  |  |  |  |  |  |  |  |  |  |  |  |  |  |  |  |  |  |  |
| **PCT3 Follow-up assessment** |  |  |  |  |  |  |  |  |  |  |  |  |  |  |  |  |  |  |  |  |  |  |  |  |  |  |  |  |  |  |  |  |  |  |  |  |
| **Focus groups** |  |  |  |  |  |  |  |  |  |  |  |  |  |  |  |  |  |  |  |  |  |  |  |  |  |  |  |  |  |  |  |  |  |  |  |  |
| **Final report** |  |  |  |  |  |  |  |  |  |  |  |  |  |  |  |  |  |  |  |  |  |  |  |  |  |  |  |  |  |  |  |  |  |  |  |  |
| **Interim report (6monthly)** |  |  |  |  |  |  |  |  |  |  |  |  |  |  |  |  |  |  |  |  |  |  |  |  |  |  |  |  |  |  |  |  |  |  |  |  |
| **Steering group (6 monthly)** |  |  |  |  |  |  |  |  |  |  |  |  |  |  |  |  |  |  |  |  |  |  |  |  |  |  |  |  |  |  |  |  |  |  |  |  |

**BENEFITS OF THIS RESEARCH**

Results from this evaluation will be of direct policy relevance to the development of diabetes services in the UK. Diabetes and service reconfiguration are both national research priorities but there is an urgent need to base policy on evidence. Modelling suggests the ICCs will be cost effective in reducing complications and consequent hospital admissions, as well as producing societal benefits such as reduced incapacity to work. If results from this short term evaluation are positive they will provide a strong evidence base for early adoption and longer term evaluations to assess the benefits predicted from modelling.

**INVOLVEMENT OF STAKEHOLDERS**

All relevant stakeholders will be included on the project steering group, including two patient representatives recruited from the Warwick Diabetes Care User group, the leads for diabetes in participating PCTs and linked hospitals, and a representative from the funders. It will have an independent chair and advise the research team at all stages, including protocol development, design of questionnaires, consent forms etc, and interpretation and dissemination of results. It will meet 6 monthly and monitor and supervise the trial towards its interim and overall objectives and review relevant information from other sources. Users have already contributed to writing the proposal.

**PLANS FOR DISSEMINATION**

Outputs will include reports targeted to clinical, research and policy communities, as well as patient organisations and will be publicised through the university press office and Leicestershire diabetes websites. We will work with SDO centrally to maximise dissemination, including the production of a four page briefing paper, and identification of champions and ‘boundary spanners’ ^29^. For example a member of our steering group (Diabetes lead for Eastern Leicester PCT) sits on the diabetes NSF

**RESOURCES**

A **trial coordinator** will be employed for 3 years. Duties will include recruitment and liaison with practices, liaison with ICC clinics, recruitment, training and supervision of study nurses, convening of meetings of PMG and SG, supervision of database, data analysis with support from University of Warwick, and drafting of interim and final reports.

A 0.8 wte **clerical/data entry post** will be needed for 3 years. Data entry will be a major task, with about 50 sets of questionnaires to be entered daily during the two 6 month periods when initial and final assessments are taking place. Other duties will include preparation of trial materials, production and mailing out of the trial newsletter, and arrangements for patients to attend their assessments.

A **10% statistician post** and a **10% health economics post (Anil Gumbar)** is needed to lead on these aspects of the trial, contribute to development of data capture instruments and contribute to the final data analysis and drafting of reports.

The **Leicester Clinical Trials Unit** (in liaison with its partner units in Sheffield and Nottingham) will provide methodological support, including randomisation and advice on database design, as well as statistical consultancy.

In addition to staff employed on the project, the **Project Management Group** will include the following (with % time on project in parentheses) **Dr Andrew Wilson** (20%) is lead investigator and will be responsible for ensuring the success of the trial. He has experience in leading community based randomised trials and is chair of the ‘strongly rated’ UHL SDO R&D board. **Dr Kamlesh Khunti** (5%) and **Dr Melanie Davies** (5%) both have expertise in conducting RCTs and screening studies in primary care. KK and MD are currently a PI’s on a number of international and national studies including ADDITION and DESMOND. KK and MD are collaborators of MRC Co-operative on the Development and Evaluation of innovative strategies for the prevention of chronic diseases in primary care. They will advise on all aspects, especially recruitment and development of the service in the Leicester PCTs. **Professor Richard Baker** (5%) has a strong track record in SDO, implementation trials, patient perspectives and continuity of care and will advise on these aspects of the trial. **Professor Jeremy Dale** (5%) and **Dr Hillary Hearnshaw** (5%) have an established expertise in research on diabetes care which includes RCTs of diabetes care interventions in primary care, and the use of questionnaires, focus groups and interviews on diabetes studies, including multi-ethnic populations. They will advise on these elements, liaise with Coventry practices, and oversee the focus groups. **Professor Sudhesh Kumar** (5%) has expertise in investigating novel approaches to delivery of care for diabetes and obesity and is a PI for a number of national and international studies. He is a member of the steering group for COUNTERWEIGHT and UK ADS studies, and will advise on trial design as well as liaising with the ICC in Coventry. **Neil Raymond** (5%) has expertise in diabetes epidemiology, plus training and experience in health technology assessment, specifically the design and conduct of cluster randomised trial (UKADS) in diabetes care evaluation. He will provide epidemiological and statistical expertise, with support from **Professor Nigel Stallard**, Chair of Medical Statistics at Warwick University. **Professor Ala Szczepura** (5%) has expertise in multi-disciplinary research and economic evaluation of complex interventions, and **Dr Anil Gumber** (10%) in health economics and ethnic health. Both will oversee the health economics aspects of the trial. The **Warwick Diabetes Care User Group** has national recognition as a lay advisory group on diabetes related research, and will contribute two members to the steering group.

**SUMMARY RESEARCH BUDGET**

| Financial Year 1/04 – 31/03 | Year 1 (£) | Year 2 (£) | Year 3 (£) | Total |
| --- | --- | --- | --- | --- |
| B. Staff | **53,853** | **55,496** | **57,618** | **166,967** |
| D. Travel & Subsistence. | **1,750** | **1,650** | **1,650** | **5,050** |
| E. Consumables | **20,600** | **13,000** | **14,550** | **48,150** |
| F. Exceptional Items |  |  |  |  |
| G. Equipment | **1,500** |  |  | **1,500** |
| SUBTOTAL |  |  |  |  |
| H. Overheads for staff only | **24,772** | **25,528** | **26,504** | **76,804** |
| **GRAND TOTAL**  **(6B+6D+6E+6F+6G+6H)** | **98,475** | **95,674** | **100,332** | **298,471** |

**REFERENCES**

1. Gaede P, Vedel P, Larsen N, Jensen GVH, Parving H-H, Pedersen O. Multifactorial intervention and cardiovascular disease in patients with type 2 diabetes. *New England Journal of Medicine* 2003;. 348:30.

2. Turner RC, Holman RR, Cull CA, Stratton IM, Matthews DR, Frighi V *et al*. Intensive blood-glucose control with sulphonylureas or insulin compared with conventional treatment and risk of complications in patients with type 2 diabetes (UKPDS 33). *Lancet* 1998;352:837-53.

3. Turner R, Holman R, Stratton I, Cull C, Frighi V, Manley S *et al*. Tight blood pressure control and risk of macrovascular and microvascular complications in type 2 diabetes: UKPDS 38. *BMJ* 1998;317:703-13.

4. Colhoun HM, Betteridge DJ, Durrington PN, Hitman GA, Neil HA, Livingstone SJ *et al*. Primary prevention of cardiovascular disease with atorvastatin in type 2 diabetes in the Collaborative Atorvastatin Diabetes Study (CARDS): multicentre randomised placebo-controlled trial.[see comment]. *Lancet* 2004;364:685-96.

5. Ahroni JH, Boyko EJ, Davignon DR, Pecoraro RE, Ahroni JH, Boyko EJ *et al*. The health and functional status of veterans with diabetes. *Diabetes Care* 1994;17:318-21.

6. Renders CM, Valk GD, Griffin S, Wagner EH, Eijk JT, Assendelft WJ *et al*. Interventions to improve the management of diabetes mellitus in primary care, outpatient and community settings. *Cochrane Database Syst.Rev.* 2001;CD001481.

7. Olivarius NDF, Beck-Nielsen H, Andreasen AH, Horder M, Pedersen PA. Randomised controlled trial of structured personal care of type 2 diabetes mellitus. *Br Med J* 2001;. 323:27.

8. Gray A, Clarke P, Farmer A, Holman R. Implementing intensive control of blood glucose concentration and blood pressure in type 2 diabetes in England: Cost analysis (UKPDS 63). *Br Med J* 2002;. 325:19.

9. Goyder EC, McNally PG, Drucquer M, Spiers N, Botha JL. Shifting of care for diabetes from secondary to primary care, 1990-5: Review of general practices. *Br Med J* 1998;316:1505-6.

10. Khunti K, Ganguli S, Baker R, Lowy A. Features of primary care associated with variations in process and outcome of care of people with diabetes. *British Journal of General Practice* 2001;51:356-60.

11. Hippisley-Cox J, O'Hanlon S, Coupland C. Association of deprivation, ethnicity, and sex with quality indicators for diabetes: population based survey of 53,000 patients in primary care. *BMJ* 2004;329:1267-9.

12. Griffin SJ. The management of diabetes. *British Medical Journal* 2001;323:946-7.

13. Nocon A,.Leese B. The role of UK general practitioners with special clinical interests: implications for policy and service delivery. *British Journal of General Practice* 2004;54:50-6.

14. Nocon A, Rhodes PJ, Wright JP, Eastham J, Williams DRR, Harrison SR *et al*. Specialist general practitioners and diabetes clinics in primary care: A qualitative and descriptive evaluation. *Diabetic Medicine* 2004;21:32-8.

15. Mainous AG, III, Koopman RJ, Gill JM, Baker R, Pearson WS. Relationship between continuity of care and diabetes control: evidence from the Third National Health and Nutrition Examination Survey. *Am.J.Public Health* 2004;94:66-70.

16. Association of Public Health Observatories (APHO) Yorkshire & Humber. PBS Diabetes Population Prevalence Model. 2004.

17. Campbell M, Fitzpatrick R, Haines A, Kinmonth AL, Sandercock P, Spiegelhalter D *et al*. Framework for design and evaluation of complex interventions to improve health. *BMJ* 2000;321:694-6.

18. Khunti K, Stone M, Luan X, Gisbourne L, Baines J, Paul S *et al*. Clinics for intermediate review and intervention by nurse specialists in cardiology (the CLINIC trial). *Abstract Book SAPC 33rd Annual Scientific Meeting*, 2004.

19. The NHS Confederation. New GMS Contract 2003: Investing in General Practice. General Practitioners Committee. 2003. London, British Medical Association.

20. Bradley F, Wiles R, Kinmonth AL, Mant D, Gantley M. Development and evaluation of complex interventions in health services research: case study of the Southampton heart integrated care project (SHIP). The SHIP Collaborative Group. *BMJ* 1999;318:711-5.

21. The Euroqol Group. EQ-5D User Guide: A measure of health-related quality of life developed by the EuroQol Group. The Euroqol Group. 16-11-1995. The Netherlands, EuroQol Business Management.

22. Bradley C. Diabetes treatment satisfaction questionnaire. In Bradley C, ed. *Handbook of Psyc Diabetes*, Chur, Switzerland: Harwood Academic Publishers, 1994.

23. Welch GW, Jacobson AM, Polonsky WH. The Problem Areas in Diabetes Scale. An evaluation of its clinical utility. *Diabetes Care* 1997;20:760-6.

24. Baker R,.Whitfield M. Measuring patient satisfaction: a test of construct validity. *Qual.Health Care* 1992;1:104-9.

25. Kinmonth AL, Woodcock A, Griffin S, Spiegal N, Campbell MJ. Randomised controlled trial of patient centred care of diabetes in general practice: impact on current wellbeing and future disease risk. *BMJ.* 1998;317:1202-8.

26. O'Hare JP, Raymond NT, Mughal S, Dodd L, Hanif W, Ahmad Y *et al*. Evaluation of delivery of enhanced diabetes care to patients of South Asian ethnicity: the United Kingdom Asian Diabetes Study (UKADS). *Diabet.Med.* 2004;21:1357-65.

27. Donner A, Klar N. Design and analysis of cluster randomization trials in health research. London: Arnold Publishers, 2000.

28. Ritchie J, spencer L. Qualitative data analysis for applied policy research. In Bryman A, Burgess G, eds. London and New York: Routledge, 1994.

29. Greenhalgh, T, Robert, G, and Bate, P. How to spread good ideas: a systematic review of the literature on diffusion, dissemination and sustainability of innovations in health service delivery and organisation (Report for the NCCSDO). University College London. 2004.

**APPENDIX 1: ESTABLISHING CAPACITY IN INTERMEDIATE CARE CLINICS**

Although participating PCTs differ in size, we plan to develop similar sized ICC in three localities (EL, LCW and Rugby/N Warks combined) Patients of practices in the intervention arm will have access to ICC whether or not they consent to the study. We have estimated that 70% of eligible patients will consent and 30% will not. To deliver our required sample size of 2550 study patients we therefore need to provide capacity for 3825 patients. Assuming that 30% of these will attend ICC over an 18 month period means that the clinics will have to have capacity for 1147 new patients over 18 months, ie 765 patients per annum, or 18 patients per week. These calculations are shown below, in total and per PCT (combining Rugby and NWarks)

|  | total | per pct (combining R and NW) |
| --- | --- | --- |
| total number of practices recruited | 51 | 17 |
| practices randomised to intervention group | 25-26 | 8 or 9 |
| patients randomised to intervention group (150 per 6000 pop practice) | 3825 | 1275 |
| agree to study (70%) | 2550 | 850 |
| Total attenders at ICC over18m (assuming 30% referred) | 1147 | 382 |
| Of whom 70% (803) will have agreed to study |  |  |
| Total attending ICC per annum | 765 | 255 |
| Total attending ICC per week (assuming 42 working weeks) | 18 | 6 |
| Assuming 3 attendances per patient, total consultations pa | 2295 | 765 |

| **TREATMENT COSTS** | **YEAR 1** | **YEAR 2** | **TOTAL** |
| --- | --- | --- | --- |
| Development and running of ICCD clinics |  |  |  |
| 8 consultant/gpwsi sessions per week (5 Leic, 3R/NW) 12.5k pa  per session | **100,000** | **50,000** | **150,000** |
| 8 specialist nurses sessions per week estimate 5k per session | **40,000** | **20,000** | **60,000** |
| 8 clinic coordinators sessions estimate 2k per session (including support costs) | **16,000** | **8,000** | **24,000** |
| **ORIGINAL BID 230,000** |  |  |  |
|  |  |  | **234,000** |

Assuming each patient is seen three times, total clinic capacity in needs to be for 18 new patients and 36 follow up patients per week, a total of 48 patients. PCTs have estimated that a half day clinic staffed by a doctor and specialist nurse with admin support could see 6 patients, meaning that for the whole study 8 half day clinics will be required. Approximate costs for this are shown below.

**APPENDIX 2: NHS SUPPORT COSTS**

NHS support costs will be sought to cover the baseline and final assessments, including nurse time and laboratory costs, as well as the extra costs for recruitment and data capture incurred by practices. Baseline and follow up assessments will take place over 8-9 months, approximately 1 month each for the practices in the catchment area of each of the 8 proposed ICCs. We have assumed that each fte nurse will undertake 6 assessments a day and will employ 3 G grade nurses and 1.5 B grade nurses for 12 months to undertake these. Consent will be confirmed by the G grade nurses. We have assumed that 70% of participants will attend follow up assessments, which will be undertaken by 3 B grade nurses over a period of 9 months.

| **NHS COSTS** | **YEAR 1** | **YEAR 2** | **TOTAL** |
| --- | --- | --- | --- |
| Reimbursement of practice costs (recruitment, data capture) £1000 each | 51,000 |  | 51,000 |
| baseline assessments (1.5 B, 3G grade) for 12m | 120,000 |  | 120,000 |
| final assessments 3B grade for 12m |  | 42,000 | 42,000 |
| Lab costs (8700@£ 14.38) | 73,338 | 51,768 | 125,106 |
| 30 GP (5 days in each PCT) sessions to assist with practice recruitment (1 session = £200) | 6,000 |  |  |
| **ORIGINAL BID 342,000** |  |  |  |
| ***subtotal*** | **250,338** | **93,768** | **344,106** |

| **NURSE COSTS** |  |
| --- | --- |
| **b/line assessments 1.8.06-31.4.07** | 5,100 |
| number of weeks (9months) | 39 |
| assessments/week | 131 |
| wte nurse time (@ 30 assessments/w) | 4.5 |
|  |  |
| **FU assessments 1.2.08-31.10.08** | 3,570 |
| number of weeks | 39 |
| assessments/week | 92 |
| wte nurse time (@ 30 assessments/w) | 3.05 |
|  |  |
| total assessments | 8,670 |
|  |  |
| 3 G grade for b/line | 96,000 |
| plus 1.5 B grade for bline | 24,000 |
| 3 B grade for follow up | 42,000 |
| total cost | 162,000 |

**APPENDIX 3: PATIENT INFORMATION SHEET**

**Patient Information Sheet (Version 2, 27/6/06)**

**1 Study title**

Models of care for diabetes in the community (MODCOM)

**2 Invitation**

You are being invited to take part in a research study. Before you decide it is important for you to understand why the research is being done and what it will involve. Please take time to read the following information carefully. Talk to others about the study if you wish. Ask us if there is anything that is not clear or if you would like more information. Take time to decide whether or not you wish to take part.

**3 What is the purpose of the study?**

There is now very good evidence that controlling blood sugar and other risk factors such as blood pressure improves the quality of life for people with diabetes as well as reducing the risk of complications such as heart attack and stroke.

In this study we are comparing two different ways of organising health care for people with diabetes. One method is for GPs and practice nurses to work with colleagues in hospital. Another is to provide community based clinics for diabetes (sometimes known as intermediate care clinics) which act as a ‘bridge’ between practices and the hospital. The aim of this study is to find out which method of care is more effective in helping patients with diabetes achieve the best control possible of their disease.

**4 Why have I been chosen?**

Your practice is taking part in this study and will soon be allocated by chance to one of the two methods of care we are comparing. The practice is inviting all its adult patients with type 2 diabetes to take part.

**5 Do I have to take part?**

No. It is up to you to decide whether or not to take part. If you do, you are still free to withdraw at any time and without giving a reason.

**6 What will happen to me if I do not take part?**

A decision not to take part will not affect the care you receive or whether your doctor suggests referral to a community clinic or hospital.

**7 What will happen to me if I take part?**

After the study has started you will be asked to attend for two assessments, the first at the beginning of the study and the second 18-24 months later. The first assessments will take place within the next few weeks and at that time a research nurse will explain the study to you in more detail and ask you to confirm your consent in writing. At both assessments the research nurse will check your weight, blood pressure and waist circumference and ask you to complete some questionnaires. You will also be asked to provide a sample of 10 ml blood (2 teaspoons) and a similar amount of urine to assess how well controlled your diabetes is. Both assessments will take place in your general practice or at your home if you are housebound. It is very important that as many people as possible who attend the first assessment also attend the second, otherwise we may not be able to tell which method of care is more effective. We will also ask you for permission to look at your medical notes to find out, for example, how many times you saw your doctor or nurse. Each assessment will take up to an hour.

At the end of the study you may be invited to participate in a more detailed interview about what you think about the care you have received. If you are selected you will receive more detailed information at the time and be asked to complete a further consent form.

**8 What will happen if I don’t want to carry on with the study?**

You can withdraw from the study at any time, and this will not affect the care you receive. If you decide not to attend the second assessment we will ask you whether you are still prepared to allow us to look at your GP records and complete a questionnaire.

**9 What are the possible disadvantages and risks of taking part?**

The only disadvantage is the time you have given up to attend the assessments, and the slight pain you may feel when giving blood.

**10 What are the possible benefits of taking part?**

We cannot promise the study will help you but the information we get might help improve the organisation of care of people with diabetes.

**11 What happens when the research study stops?**

There will be no immediate change in the way your practice organises its care for people with diabetes after the trial has stopped. In the longer term results may influence how care is organised.

12 Expenses and payments:

You will be able to claim for any additional expenses incurred through attending the assessments.

**13 Will my taking part in the study be kept confidential?**

Yes. All information which is collected about you during the course of the research will be kept strictly confidential. Any information about you which leaves the practice will have your name and address removed so that you cannot be recognised from it.

**14 Complaints**:

If you have a concern about any aspect of this study, you should ask to speak with the researchers who will do their best to answer your questions. If you remain unhappy and wish to complain formally, you can do this through the NHS Complaints Procedure

**15 Harm**:

In the event that something does go wrong and you are harmed during the research study there are no special compensation arrangements. If you are harmed and this is due to someone’s negligence then you may have grounds for a legal action for compensation against the University of Leicester but you may have to pay your legal costs. The normal National Health Service complaints mechanisms will still be available to you. NHS and University Indemnity do not offer no-fault compensation i.e. for non-negligent harm, and NHS bodies are unable to agree in advance to pay compensation for non-negligent harm*.*

16 What will happen to any samples I give?

The blood samples taken as part of the research will be given a code number before they leave the practice so that only members of the research team will be able to link them with you. Your doctor will not be given the results of your first assessment, which is for research purposes only. However the results of your second assessment will, with your permission, be given to your doctor and will contribute to your ongoing care. Samples will be destroyed after analysis, according to the laboratory’s usual practice.

**17 What will happen to the results of the research study?**

Results will be presented to NHS managers to help them decide how to arrange care. Findings will also be written for publication in scientific journals and presented at conferences. If you are interested in knowing the results you will be able to see them on the study’s website.

**18 Who is organising and funding the research?**

The study if funded by the NHS Service Delivery and Organisation research and development programme. Your doctor will be paid only for the additional costs incurred by participating in the study

**19 Contact Details:**

The chief investigator is Dr Andrew Wilson, Department of Health Sciences, University of Leicester, Leicester General Hospital, Leicester LE5 4PW

Tel 0116 258 4367

aw7@le.ac.uk

**If you decide to participate you will be given a copy of this information sheet and a signed consent form to keep. Thank you for considering taking part.**

**CONSENT FORM (Version 2, 27/06/06)**

***(Form to be on headed paper, to be completed at time of first assessment)***

**Title of Project: Models of care for diabetes in the community (MODCOM)**

**Name of Researcher: Andrew Wilson**

Centre Number:

Patient Identification Number:

**Please initial box**

1. I confirm that I have read and understand the information sheet dated 27/06/2006 (version 2) for the above study. I have had the opportunity to consider the information, ask questions and have had these answered satisfactorily.

2. I understand that my participation is voluntary and that I am free to withdraw at any time, without giving any reason, without my medical care or legal rights being affected.

3. I understand that relevant sections of any of my medical notes and data collected during the study, may be looked at by responsible individuals from the University of Leicester, from regulatory authorities or from the NHS Trust, where it is relevant to my taking part in this research. I give permission for these individuals to have access to my records.

4. I agree to take part in the above study.

________________________ ________________ ________________

Name of Patient Date Signature

_________________________ ____________________ ________________

Name of Person taking consent Date Signature

(if different from researcher)

_________________________ ____________________ ________________

Researcher Date Signature

When completed, 1 for patient; 1 for researcher site file; 1 (original) to be kept in GP notes

**APPENDIX 5: GP INVITATION LETTER**

**GP invitation letter version 1, 23/2/06**

Dear …………….

**TRIAL OF INTERMEDIATE CARE CLINICS FOR DIABETES (ICCD)**

An intermediate care clinic for diabetes will soon be set up in your locality. *(details of local arrangements)* We have been funded by the NHS Service Development and Organisation (SDO) programme to evaluate the effectiveness of these clinics and are writing to all practices in the locality to invite them to take part.

***What is the aim of the clinics?***

Clinics will be staffed by a multi-disciplinary diabetes team to provide short term support to GPs and practice nurses in managing patients with type 2 diabetes with whom you are having difficulty achieving optimal metabolic control. We expect substantial variation between practices in the extent to which they use the new service but an average practice will probably refer about 20% of their patients with diabetes over a 12 month period.

***What is the aim of the trial?***

The trial will compare the metabolic control of all patients with type 2 diabetes in practices which have access to the clinics with patients of practices that do not have access. Practices not taking part in the trial will not have access to the new clinics.

***What will the trial involve for my practice?***

Practices which agree to take part will be randomised so that half will have access to the clinic, while the remainder continue with their current management during the 18 month period of the trial. We need at least 50% of practices in each locality to make the study viable. If more practices than needed wish to participate we will give priority to those with lower QoF scores for diabetes.

***What will the trial involve for my patients with diabetes?***

All your patients with type 2 diabetes will be invited to attend a baseline assessment with a research nurse at the start of the study and a similar follow up assessment 18-24 months later. Results (including BMI, blood pressure HbA1 and lipids) from the second assessment will be made available to you and so contribute to patients’ annual reviews and your QoF targets. These assessments will take place in your practice at a time agreed with you and will include examination of the medical record. All your patients with type 2 diabetes will have access to the intermediate care clinics if your practice is in the intervention arm, whether or not they consent to take part in the study.

***When will practices be randomised?***

Practices will be randomised to intervention and control groups after the baseline assessments have been completed.

***What are the advantages of taking part?***

Advantages include the provision of data that contribute to your annual reviews of patients with diabetes and a 50% chance of accessing the new service. A fee of £1000 will be payable to both intervention and control practices for use of their premises and access to patient records.

We hope your practice will agree to take part in this important study. Please indicate on the attached slip whether you are interested in learning more about the trial, in which case we will arrange a visit to discuss it in more detail.

Yours sincerely

***AW, KK/AF/MD/SK***

***Reply slip***

**Practice name**

**Our practice is/is not interested in participating in this trial.**

**Please return in the stamped addressed envelope provided.**

**APPENDIX 6: PATIENT ASSESSMENT PACK**

**MODCOM STUDY: PATIENT ASSESSMENTS**

**Practice Code:**

**Patient Code:**

**Contents:**

**1. Demographic (initial assessment only) and medical data**

**2. Examination**

**3. Questionnaires - Continuity questionnaire (interviewer)**

**- WHOQoL (this and subsequent questionnaires self administered)**

**- PAID**

**- DTSQ**

**- EQ5D**

**- Patient costs questionnaire (final assessment only, will be developed during study)**

**DEMOGRAPHIC AND MEDICAL DATA (interviewer administered)**

| Date of Birth: |  |
| --- | --- |
|  |  |
| Sex | M F |
|  |  |
| Ethnicity | White British  White Irish  Indian  Pakistani  Bangladeshi  Black Caribbean  Black African  Chinese  Other |
|  |  |
| Did your education continue after the  minimum school leaving age? | YES NO |
| Do you have a degree or equivalent qualification | YES NO |
|  |  |
| Which of the following best describes your main activity? | In employment or self employment  Retired  Housework  Student  Seeking work  Unable to work due to ill health  Other (please state) |
|  |  |
| Do you live alone? | YES NO |
| If no do you live with the following | Husband/wife/partner  Children under 18  Son/daughter 18 or over  Parents/parents in law/step-parents  Other family or friends |
|  |  |
| Are you a current smoker? | YES NO |
| If Yes, number of cigarettes/day |  |
|  |  |
| Co-morbidities (From GP record) |  |
| - Hypertension | YES NO |
| - Ischaemic heart disease | YES NO |
| - Cerebrovascular disease | YES NO |
| - Heart failure | YES NO |
| - Peripheral vascular disease | YES NO |
| - Renal failure | YES NO |
| Current medication (printout) |  |
|  |  |
| Was interpreter/ translated materials needed |  |
| If yes state language |  |

**EXAMINATION**

| Height |  |
| --- | --- |
| Weight |  |
| BMI |  |
| Waist circumference |  |
| Blood pressure |  |
| Proteinuria |  |
| Bloods taken (tick) | HbA_1_C |
|  | Glucose |
|  | Lipids |
|  |  |
| Is patient fasting? | YES NO |

#### CONTINUITY QUESTIONNAIRE (Interviewer administered)

#### *These questions ask about your diabetes care from the general practice (surgery)*

**LC1-gp** In the last 12 months, how many times have you spoken with staff at the **practice** about your diabetes?

*Never*  *^0^*

*If you answered ‘never’ go on to question TCB1*

*Once*  *^1^*

*Twice*

*3 times*  *^3^*

*4 times*  *^4^*

*5 times or more*  *^5^*

**LC2-gp** In the past 12 months, how many times has the **practice** sent you an appointment letter for your diabetes?

*Never*  *^0^*

*Once*  *^1^*

*Twice*  *^2^*

*3 times*  *^3^*

*4 times*  *^4^*

*5 times or more*  ^5^

**LC3-gp** In the past 12 months, how many times have you had a blood test (including fingerprick tests) taken for your diabetes at the **practice**?

*Never*  *^0^*

*Once*  *^1^*

*Twice*  *^2^*

*3 times*  *^3^*

*4 times*  *^4^*

*5 times or more*  *^5^*

**FC1-gp** If you need advice **urgently,** how long does it take to get to speak to a doctor or nurse at the **practice**?

*Same day*  *^5^*

*Next working day*  *^4^*

*Within 2 working days*  *^3^*

*Within 3 working days*  *^2^*

*Within 4 working days*  *^1^*

*In 5 working days or more*  *^0^*

*I don’t know*  *^8^*

*I don’t go to the GP for emergencies*  *^9^*

*If this question is not applicable, go to question FC3-gp*

FC2-gp **How would you rate the length of time you’ve had to wait before you spoke to a doctor or nurse at the** practice, **if you needed urgent advice?**

*Very poor*  *^0^*

*Poor*  *^1^*

*Fair*  *^2^*

*Good*  *^3^*

*Very good*  *^4^*

***Excellent*** *^5^*

FC3-gp **If you have a problem with your diabetes, how well does your** practice **respond to it?**

*Extremely badly*  *^0^*

*Very badly*  *^1^*

*Badly*  *^2^*

*Fairly well*  *^3^*

*Very well*  *^4^*

*Extremely well*  *^5^*

***The next questions are about your usual doctor or nurse at the general practice. That is the doctor or nurse who knows you and your diabetes best.***

**LC4-gp** In the last 12 months, how many times have you seen your **usual** doctor or nurse at the **practice**?

*I don’t have a usual doctor or nurse*  *^0^*

*Once*  *^1^*

*Twice*  *^2^*

*3 times*  *^3^*

*4 times*  *^4^*

*5 times or more*  *^5^*

LC4 a) Can I just check, does that mean there is no particular doctor or nurse at the practice who knows you and your diabetes best?

No usual doctor or nurse  ^0^

*If you do not have a usual*  *^1^ doctor or nurse go on to question TCB1*

LC4 b) Who do you usually see for your diabetes care (who

knows you and your diabetes best)?

*Practice Doctor*  *1*

*Practice Nurse*  *2*

*Both*   3

**FC4-gp** If you need to speak to your **usual** doctor or nurse about your diabetes, how easy is it for you to speak to your usual doctor or nurse at the **practice**?

*Extremely difficult*  *^0^*

*Very difficult*  *^1^*

*Somewhat difficult*  *^2^*

*Fairly easy*  *^3^*

*Very easy*  *^4^*

*Extremely easy*  *^5^*

**RC1-gp** How well does your **usual** doctor or nurse at the **practice** explain medical procedures and tests done for your diabetes?

*Extremely badly*  *^0^*

*Very badly*  *^1^*

*Badly*  *^2^*

*Fairly well*  *^3^*

*Very well*  *^4^*

*Extremely well*  *^5^*

How much do you disagree or agree with the following statements about your usual doctor or nurse at the practice?

**RC2-gp** My usual doctor or nurse at the practice **involves me in decisions** about my diabetes.

*Very strongly disagree*  *^0^*

*Strongly disagree*  *^1^*

*Disagree*  *^2^*

*Agree*  *^3^*

*Strongly agree*  *^4^*

*Very strongly agree*  *^5^*

**RC3-gp** My usual doctor or nurse at the practice **listens to what I have to say.**

*Very strongly disagree*  *^0^*

*Strongly disagree*  *^1^*

*Disagree*  *^2^*

*Agree*  *^3^*

*Strongly agree*  *^4^*

*Very strongly agree*  *^5^*

**RC4-gp** My usual doctor or nurse at the practice **knows about my medical history** (e.g. other illnesses and treatments).

*Very strongly disagree*  *^0^*

*Strongly disagree*  *^1^*

*Disagree*  *^2^*

*Agree*  *^3^*

*Strongly agree*  *^4^*

*Very strongly agree*  *^5^*

**RC5-gp** My usual doctor or nurse at the practice **makes the best decisions about my diabetes treatment** (e.g. medication, tests done).

*Very strongly disagree*  *^0^*

*Strongly disagree*  *^1^*

*Disagree*  *^2^*

*Agree*  *^3^*

*Strongly agree*  *^4^*

*Very strongly agree*  *^5^*

**RC6-gp** My usual doctor or nurse at the practice **is concerned about me.**

*Very strongly disagree*  *^0^*

*Strongly disagree*  *^1^*

*Disagree*  *^2^*

*Agree*  *^3^*

*Strongly agree*  *^4^*

*Very strongly agree*  *^5^*

This section is about your overall experience of diabetes care (Practice and other care).

TCB1 **In general, how well is your diabetes care co-ordinated?**

*Extremely badly*  *^0^*

*Very badly*  *^1^*

*Badly*  *^2^*

*Fairly well*  *^3^*

*Very well*  *^4^*

***Extremely well*** *^5^*

Think about all the different staff involved in your diabetes care, how much would you agree with the following statements:

TCB2  **They all give me the same information and advice.**

*Very strongly disagree*  *^0^*

*Strongly disagree*  *^1^*

*Disagree*  *^2^*

*Agree*  *^3^*

*Strongly agree*  *^4^*

*Very strongly agree*  *^5^*

**TCB3** They all know my medical history (eg other illnesses and treatments).

*Very strongly disagree*  *^0^*

*Strongly disagree*  *^1^*

*Disagree*  *^2^*

*Agree*  *^3^*

*Strongly agree*  *^4^*

*Very strongly agree*  *^5^*

**TCB4** They all know about my diabetes treatment (eg medication, tests done)

*Very strongly disagree*  *^0^*

*Strongly disagree*  *^1^*

*Disagree*  *^2^*

*Agree*  *^3^*

*Strongly agree*  *^4^*

*Very strongly agree*  *^5^*

**TCB5** They share an agreed plan of treatment for my diabetes.

*Very strongly disagree*  *^0^*

*Strongly disagree*  *^1^*

*Disagree*  *^2^*

*Agree*  *^3^*

*Strongly agree*  *^4^*

*Very strongly agree*  *^5^*

WHOQOL–BREF

UK VERSION


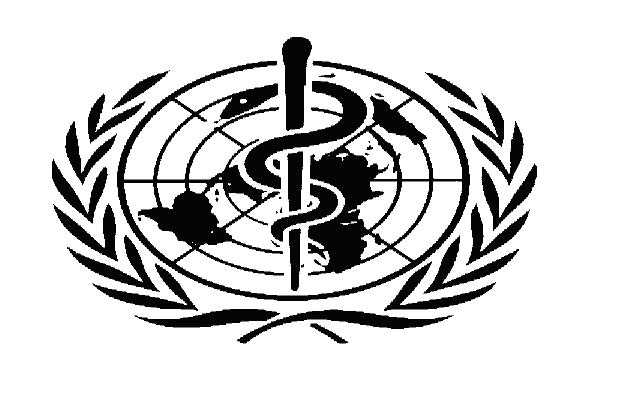


Department of Mental Health

World Health Organisation

Geneva

For Office Use Only

|  | Equations for computing domain scores | Raw score | Transformed score | |
| --- | --- | --- | --- | --- |
|  |  |  | 4­20 | 0­100 |
| Domain 1 | (6­Q3) + (6­Q4) + Q10 + Q15 + Q16 + Q17 + Q18   +  +  +  +  +  +  | = |  |  |
| Domain 2 | Q5 + Q6 + Q7 + Q11 + Q19 + (6­Q26)   +  +  +  +  +  | = |  |  |
| Domain 3 | Q20 + Q21 + Q22   +  +  | = |  |  |
| Domain 4 | Q8 + Q9 + Q12 + Q13 + Q14 + Q23 + Q24 + Q25   +  +  +  +  +  +  +  | = |  |  |

This document is not issued to the general public and all rights are reserved by the World Health Organisation (WHO). This document may not be reviewed, abstracted, quoted, reproduced, translated, referred to in bibliographic matter or cited in part or in whole without prior written permission of the WHO. No part of this document may be stored in a retrieval system or transmitted in any form by any means – electronic, mechanical or other – without the prior written permission of the WHO. The WHOQOL Group, Department of Mental Health, WHO, CH­1211, Geneva 27, Switzerland. Permission to use the UK instrument must be obtained from Professor Suzanne Skevington, WHO Centre for the Study of Quality of Life, University of Bath, Bath, BA2 7AY, UK (s.m.skevington@bath.ac.uk)

1

**Instructions**

This questionnaire asks how you feel about your quality of life, health and other areas of your life. **Please answer all the questions**. If you are unsure about which response to give to a question, **please choose the ONE** that appears most appropriate. This can often be your first response.

Please keep in mind your standards, hopes, pleasures and concerns. We ask that you think about your life **in the last two weeks**. For example, thinking about the last two weeks, a question might ask:

|  |  | Not at all | Not much | Moderately | A great deal | Completely |
| --- | --- | --- | --- | --- | --- | --- |
|  | Do you get the kind of support from others that you need? | 1 | 2 | 3 | 4 | 5 |

|  |  | Not at all | Not much | Moderately | A great deal | Completely |
| --- | --- | --- | --- | --- | --- | --- |
|  | Do you get the kind of support from others that you need? | 1 | 2 | 3 | 4 | 5 |

You should **circle** the number that best fits how much support you got from others **over the last two weeks**. So you would circle the number 4 if you got a great deal of support from others as follows:

You would circle the number 1 if you did not get any of the support that you needed from others in the last two weeks. Please read each question, assess you feelings, and **circle** the number on the scale for each question that gives the best answer for you.

2

|  |  | Very poor | Poor | Neither poor nor good | Good | Very good |
| --- | --- | --- | --- | --- | --- | --- |
| 1 | How would you rate your quality of life? | 1 | 2 | 3 | 4 | 5 |

|  |  | Very Dissatisfied | Dissatisfied | Neither Satisfied nor Dissatisfied | Satisfied | Very Satisfied |
| --- | --- | --- | --- | --- | --- | --- |
| 2 | How satisfied are you with your health? | 1 | 2 | 3 | 4 | 5 |

The following questions ask about **how much** you have experienced certain things **in the last two weeks**.

|  |  | Not at all | A little | A moderate amount | Very much | An extreme amount |
| --- | --- | --- | --- | --- | --- | --- |
| 3 | How much do you feel that pain prevents you from doing what you need to do? | 1 | 2 | 3 | 4 | 5 |
| 4 | How much do you need medical treatment to function in your daily life? | 1 | 2 | 3 | 4 | 5 |
| 5 | How much do you enjoy life? | 1 | 2 | 3 | 4 | 5 |

|  |  | Not at all | A little | A moderate amount | Very much | Extremely |
| --- | --- | --- | --- | --- | --- | --- |
| 6 | To what extent do you feel life to be meaningful? | 1 | 2 | 3 | 4 | 5 |
| 7 | How well are you able to concentrate? | 1 | 2 | 3 | 4 | 5 |
| 8 | How safe do you feel in your daily life? | 1 | 2 | 3 | 4 | 5 |
| 9 | How healthy is your physical environment? | 1 | 2 | 3 | 4 | 5 |

The following questions ask about **how completely** you experience or were able to do certain things **in the last two weeks**.

|  |  | Not at all | A little | Moderately | Mostly | Completely |
| --- | --- | --- | --- | --- | --- | --- |
| 10 | Do you have enough energy for everyday life? | 1 | 2 | 3 | 4 | 5 |
| 11 | Are you able to accept your bodily appearance? | 1 | 2 | 3 | 4 | 5 |
| 12 | To what extent do you have enough money to meet your needs? | 1 | 2 | 3 | 4 | 5 |
| 13 | How available to you is the information that you need in your day­to­day life? | 1 | 2 | 3 | 4 | 5 |
| 14 | To what extent do you have the opportunity for leisure activities? | 1 | 2 | 3 | 4 | 5 |

3

The following questions ask you to say **how good or satisfied** you have felt about various aspects of your life **over the last two weeks**.

|  |  | Very poor | Poor | Neither poor nor good | Good | Very good |
| --- | --- | --- | --- | --- | --- | --- |
| 15 | How well are you able to get around? | 1 | 2 | 3 | 4 | 5 |

|  |  | Very dissatisfied | Dissatisfied | Neither satisfied nor dissatisfied | Satisfied | Very satisfied |
| --- | --- | --- | --- | --- | --- | --- |
| 16 | How satisfied are you with your sleep? | 1 | 2 | 3 | 4 | 5 |
| 17 | How satisfied are you with your ability to perform daily living activities? | 1 | 2 | 3 | 4 | 5 |
| 18 | How satisfied are you with your capacity for work? | 1 | 2 | 3 | 4 | 5 |
| 19 | How satisfied are you with yourself? | 1 | 2 | 3 | 4 | 5 |
| 20 | How satisfied are you with your personal relationships? | 1 | 2 | 3 | 4 | 5 |
| 21 | How satisfied are you with your sex life? | 1 | 2 | 3 | 4 | 5 |
| 22 | How satisfied are you with the support you get from your friends? | 1 | 2 | 3 | 4 | 5 |
| 23 | How satisfied are you with the conditions of your living place? | 1 | 2 | 3 | 4 | 5 |
| 24 | How satisfied are you with your access to health services? | 1 | 2 | 3 | 4 | 5 |
| 25 | How satisfied are you with your transport? | 1 | 2 | 3 | 4 | 5 |

The following question refers to **how often** you have felt or experienced certain things **in the last two weeks**.

|  |  | Never | Seldom | Quite often | Very often | Always |
| --- | --- | --- | --- | --- | --- | --- |
| 26 | How often do you have negative feelings, such as blue mood, despair, anxiety, depression? | 1 | 2 | 3 | 4 | 5 |

Did someone help you to fill out this form? **YES / NO**

**Problem Areas in Diabetes (PAID)**

Which of the following diabetes issues are currently problems for you? Please circle the number that gives the best answer for you. Please provide an answer for each question.

|  | **Not a Problem** | **Minor Problem** | **Moderate Problem** | **Somewhat Serious Problem** | **Serious Problem** |
| --- | --- | --- | --- | --- | --- |
| Not having clear and concrete goals for your diabetes care? | **0** | **1** | **2** | **3** | **4** |
| Feeling discouraged with your diabetes treatment plan? | **0** | **1** | **2** | **3** | **4** |
| Feeling scared when you think about living with diabetes? | **0** | **1** | **2** | **3** | **4** |
| Uncomfortable social situations related to your diabetes care (e.g. people telling you what to eat)? | **0** | **1** | **2** | **3** | **4** |
| Feelings of deprivation regarding food and meals? | **0** | **1** | **2** | **3** | **4** |
| Feeling depressed when you think about living with diabetes? | **0** | **1** | **2** | **3** | **4** |
| Not knowing if your mood or feelings are related to your diabetes? | **0** | **1** | **2** | **3** | **4** |
| Feeling overwhelmed by your diabetes? | **0** | **1** | **2** | **3** | **4** |
| Worrying about low blood sugar reactions? | **0** | **1** | **2** | **3** | **4** |
| Feeling angry when you think about living with diabetes? | **0** | **1** | **2** | **3** | **4** |
| Feeling constantly concerned about food and eating? | **0** | **1** | **2** | **3** | **4** |
| Worrying about the future and the possibility of serious complications | **0** | **1** | **2** | **3** | **4** |
| Feelings of guilt or anxiety when you get off track with your diabetes management | **0** | **1** | **2** | **3** | **4** |
| Not “accepting” your diabetes | **0** | **1** | **2** | **3** | **4** |
| Feeling unsatisfied with your diabetes physician | **0** | **1** | **2** | **3** | **4** |
| Feeling that diabetes is taking up too much of your mental and physical energy every day? | **0** | **1** | **2** | **3** | **4** |
| Feeling alone with your diabetes? | **0** | **1** | **2** | **3** | **4** |
| Feeling that your friends and family are not supportive of your diabetes management efforts? | **0** | **1** | **2** | **3** | **4** |
| Coping with complications of diabetes? | **0** | **1** | **2** | **3** | **4** |
| Feeling burned out by the constant effort needed to manage diabetes? | **0** | **1** | **2** | **3** | **4** |

**The Diabetes Treatment Satisfaction Questionnaire: DTSQ**

The following questions are concerned with the treatment for your diabetes (including insulin, tablets and/or diet) and your experience over the past few **weeks.** Please answer each question by circling a number on each of the scales.

1 How satisfied are you with your current treatment?

| Very satisfied | 6 | 5 | 4 | 3 | 2 | 1 | 0 | Very dissatisfied |
| --- | --- | --- | --- | --- | --- | --- | --- | --- |

2 How often have you felt that your blood sugars have been unacceptably high recently?

| Most of the time | 6 | 5 | 4 | 3 | 2 | 1 | 0 | None of the time |
| --- | --- | --- | --- | --- | --- | --- | --- | --- |

3 How often have you felt that your blood sugars have been unacceptably low recently?

| Most of the time | 6 | 5 | 4 | 3 | 2 | 1 | 0 | None of the time |
| --- | --- | --- | --- | --- | --- | --- | --- | --- |

4 How convenient have you been finding your treatment to be recently?

| Very convenient | 6 | 5 | 4 | 3 | 2 | 1 | 0 | Very inconvenient |
| --- | --- | --- | --- | --- | --- | --- | --- | --- |

5 How flexible have you been finding your treatment to be recently?

| Very flexible | 6 | 5 | 4 | 3 | 2 | 1 | 0 | Very inflexible |
| --- | --- | --- | --- | --- | --- | --- | --- | --- |

1. How satisfied are you with your understanding of your diabetes?

| Very satisfied | 6 | 5 | 4 | 3 | 2 | 1 | 0 | Very dissatisfied |
| --- | --- | --- | --- | --- | --- | --- | --- | --- |

1. Would you recommend this form of treatment to someone else with your kind of diabetes?

| Yes, I would definitely recommend the treatment | 6 | 5 | 4 | 3 | 2 | 1 | 0 | No, I would definitely not recommend the treatment |
| --- | --- | --- | --- | --- | --- | --- | --- | --- |

1. How satisfied would you be to continue with your present form of treatment?

| Very satisfied | 6 | 5 | 4 | 3 | 2 | 1 | 0 | Very dissatisfied |
| --- | --- | --- | --- | --- | --- | --- | --- | --- |

**Please make sure you have circled only one number on each of the scales.**

Thank you for your help.

**EQ5D**

**By placing a tick in one box in each group below, please indicate which statements best describe your own health state today.**

**Mobility**

**I have no problems in walking about
I have some problems in walking about
I am confined to bed**

**Self-care**

**I have no problems with self-care
I have some problems washing or dressing myself
I am unable to wash or dress myself**

**Usual Activities (e.g. work, study, housework,
family or leisure activities)**

I have no problems with performing my usual activities
I have some problems with performing my usual activities
I am unable to perform my usual activities

**Pain/Discomfort**

**I have no pain or discomfort
I have moderate pain or discomfort
I have extreme pain or discomfort**

**Anxiety/Depression**

I am not anxious or depressed
I am moderately anxious or depressed
I am extremely anxious or depressed

**To help people say how good or bad a health state is, we have drawn a scale (rather like a thermometer) on which the best state you can imagine is marked 100 and the worst state you can imagine is marked 0.**

**Best imaginable health state**


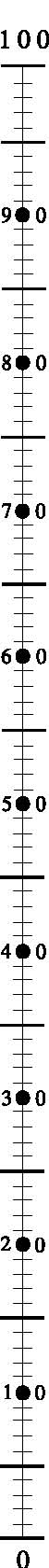


**We would like you to indicate on this scale how good or bad your own health is today, in your opinion. Please do this by drawing a line from the box below to whichever point on the scale indicates how good or bad your health state is today.**

**Your own health state today**

**Worst imaginable health state**
